# The IRE1**α**/XBP1 pathway sustains cytokine responses of group 3 innate lymphoid cells in inflammatory bowel disease

**DOI:** 10.1172/JCI174198

**Published:** 2024-05-09

**Authors:** Siyan Cao, Jose L. Fachi, Kaiming Ma, Alina Ulezko Antonova, Qianli Wang, Zhangying Cai, Randal J. Kaufman, Matthew A. Ciorba, Parakkal Deepak, Marco Colonna

**Affiliations:** 1 Division of Gastroenterology, Department of Medicine and; 2 Department of Pathology and Immunology, Washington University School of Medicine, St. Louis, Missouri, USA.; 3 Degenerative Diseases Program, Center for Genetic Disorders and Aging Research, Sanford Burnham Prebys Medical Discovery Institute, La Jolla, California, USA.

**Keywords:** Gastroenterology, Immunology, Cell stress, Inflammatory bowel disease, Innate immunity

## Abstract

Group 3 innate lymphoid cells (ILC3s) are key players in intestinal homeostasis. ER stress is linked to inflammatory bowel disease (IBD). Here, we used cell culture, mouse models, and human specimens to determine whether ER stress in ILC3s affects IBD pathophysiology. We show that mouse intestinal ILC3s exhibited a 24-hour rhythmic expression pattern of the master ER stress response regulator inositol-requiring kinase 1α/X-box–binding protein 1 (IRE1α/XBP1). Proinflammatory cytokine IL-23 selectively stimulated IRE1α/XBP1 in mouse ILC3s through mitochondrial ROS (mtROS). IRE1α/XBP1 was activated in ILC3s from mice exposed to experimental colitis and in inflamed human IBD specimens. Mice with *Ire1α* deletion in ILC3s (*Ire1α^ΔRorc^*) showed reduced expression of the ER stress response and cytokine genes including *Il22* in ILC3s and were highly vulnerable to infections and colitis. Administration of IL-22 counteracted their colitis susceptibility. In human ILC3s, IRE1 inhibitors suppressed cytokine production, which was upregulated by an IRE1 activator. Moreover, the frequencies of intestinal XBP1s^+^ ILC3s in patients with Crohn’s disease before administration of ustekinumab, an anti-IL-12/IL-23 antibody, positively correlated with the response to treatment. We demonstrate that a noncanonical mtROS-IRE1α/XBP1 pathway augmented cytokine production by ILC3s and identify XBP1s^+^ ILC3s as a potential biomarker for predicting the response to anti–IL-23 therapies in IBD.

## Abstract 2

The IRE1α/XBP1 stress pathway augments cytokine production from group 3 innate lymphoid cells and may indicate responsiveness to anti–IL-23 therapies in patients with Crohn’s disease.

## Graphical abstract

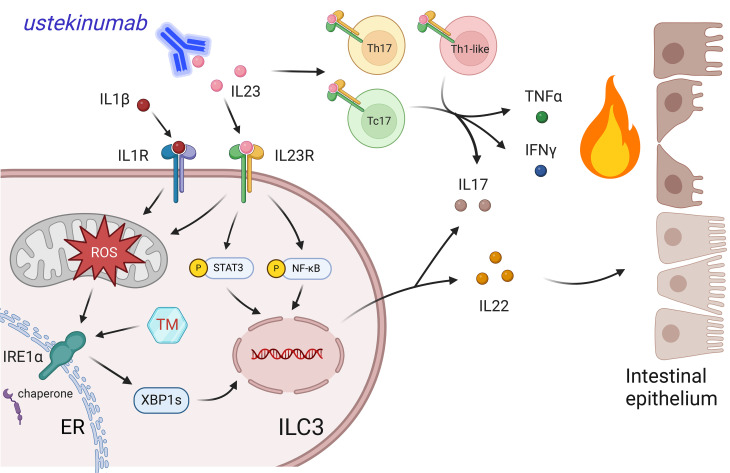

## Introduction

Innate lymphoid cells (ILCs) are a group of immune cells that exhibit lymphoid characteristics yet lack antigen-specific receptors found on T and B cells (1). Group 3 ILCs (ILC3s) comprise the most abundant ILC population in the intestine. ILC3s require the master transcription factor RORγt for their development and secrete IL-22, IL-17, and, in humans only, IL-26. Hence, ILC3s are the innate counterparts of Th17 cells. ILC3 cytokine secretion follows a circadian oscillation (2, 3) and is stimulated by IL-23, IL-1β, and IL-18, which are released by intestinal epithelial and myeloid cells in response to intestinal bacteria, nutritional components, and other environmental stimuli. Through secretion of IL-22, IL-17, and IL-26, ILC3s sustain the protective functions of epithelial, stromal, and myeloid cells, heightening epithelial barrier integrity. They also maintain intestinal tolerance to microbiota and prevent autoimmunity through induction of microbiome-specific Tregs (4, 5). Accordingly, several studies reported that ILC3s are diminished in inflamed tissue of inflammatory bowel disease (IBD) (6, 7). However, inappropriate activation of ILC3s can also promote inflammation and contribute to the physiopathology of IBD. Supporting this observation, IBD risk alleles, such as *RORC*, *IL23R*, *IL22*, *IL17A*, and *IL26* (8, 9), are not only expressed in pathogenic Th17 cells but also in ILC3s. Because of the potential relevance of ILC3s in human IBD, it is essential to fully determine the regulatory mechanisms controlling ILC3s in intestinal inflammation.

In eukaryotic cells, the ER plays a crucial role in folding and posttranslational modifications of all membrane proteins and most secreted proteins (10). This intricate process is highly sensitive to changes in both the cell’s internal and external environments. When disturbances occur, leading to the accumulation of unfolded or misfolded proteins within the ER lumen, a condition known as ER stress arises. ER stress triggers a complex cellular response called the unfolded protein response (UPR), which regulates various aspects of cell function, proliferation, and survival. In mammalian cells, ER stress activates 3 main protein sensors located on the ER membrane, namely inositol-requiring kinase 1 (IRE1), pancreatic ER eIF2α kinase (PERK), and activating transcription factor 6 (ATF6). Among these sensors, IRE1α stands out as the most conserved, possessing both a serine/threonine kinase domain and an endoribonuclease domain within its cytosolic portion. Upon activation, IRE1α cleaves a specific mRNA known as X-box–binding protein 1 (XBP1), generating a spliced isoform (XBP1s). This XBP1s protein acts as a potent transcription factor, regulating numerous cellular processes including mRNA translation within the ER, protein folding and maturation, immune and inflammatory responses, as well as antiapoptotic signaling (10–12).

Numerous stimuli induce ER stress in the gastrointestinal tract, affecting key processes in immune and nonimmune cells (13–22). Furthermore, *XBP1* variants including nonsynonymous SNPs are associated with IBD (13). Whether ER stress influences ILC3 function has not, to our knowledge, been investigated. Here, we found that mouse intestinal ILC3s expressed IRE1α and that its downstream product *Xbp1s* followed a robust circadian rhythm, which paralleled that of *Il22* at steady state. The IRE1α/XBP1 pathway was activated ex vivo in response to stimulation with the neuropeptide vasoactive intestinal peptide (VIP) and the inflammatory cytokines IL-23 and IL-1β; such activation relied on mitochondrial ROS (mtROS). IRE1α/XBP1 activation in ILC3s occurred in mice exposed to dextran sulfate sodium–induced (DSS-induced) colitis, while intestinal specimens from patients with IBD expressed higher levels of XBP1s than did healthy controls. Using a conditional-KO mouse model in which *Ire1α* was deleted in ILC3s (*Ire1α^ΔRorc^* mice), we showed that mouse ILC3s required IRE1α/XBP1 for efficient production of IL-22 and IL-17. *Ire1α^ΔRorc^* mice were highly susceptible to colitis induced by *C*. *difficile* and *C*. *rodentium* as well as to DSS-induced colitis; the latter was attenuated by administration of exogenous IL-22. In humans, cytokine production by ILC3s was attenuated by selective inhibitors of IRE1; conversely, an IRE1 activator potentiated ILC3 production of IL-22 in response to a very low concentration of IL-23. Finally, the pretreatment frequency of XBP1s-expressing ILC3s in the intestine from patients with Crohn’s disease (CD) positively correlated with their response to ustekinumab, an antagonist of the p40 subunit of IL-12/IL-23. We conclude that the IRE1α/XBP1 pathway sustains ILC3 production of cytokines that maintain intestinal homeostasis. Since XBP1s activation in ILC3s is an indicator of the responsiveness to IL-23 in patients with IBD, XBP1s^+^ ILC3s may be a useful biomarker to select a subset of patients with IBD who may benefit from therapies that inhibit IL-23.

## Results

### Intestinal ILC3s exhibit high Ire1α expression and circadian oscillation of Xbp1s.

**Figure 1:**
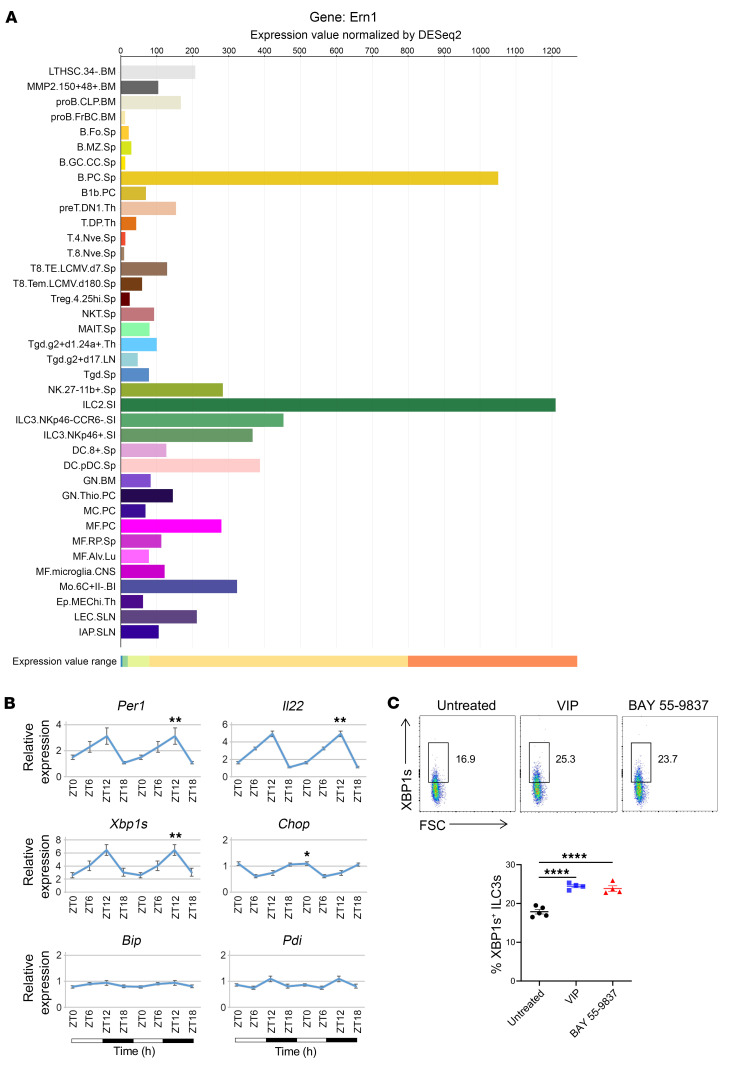
Intestinal ILC3s exhibit high *Ire1α* (*Ern1*) expression and circadian oscillation of *Xbp1s*. (**A**) Mouse intestinal ILC3s express high levels of *Ire1α* (*Ern1*). Graph shows the expression of *Ern1* in mouse immune cells from ImmGen, Gene Skyline (http://rstats.immgen.org/Skyline/skyline.html). (**B**) Gene expression (relative to β-actin) in sorted mouse siLP ILC3s (all subsets included) over a 24-hour period by qPCR. Statistical analysis was performed using MetaCycle indicated by the PJTK_CYCLE value. *n* = 8 per time point. ZT0 (6:00 am), denotes the time when the lights were turned on; ZT12 (6:00 pm), denotes the time when the lights were turned off. (**C**) Sorted mouse siLP ILC3s were treated with VIP and BAY 55-9837 (both were 1 μM) for 4 hours. Intracellular XBP1s was measured by flow cytometry. FSC, forward scatter. Error bars indicate the SEM. *P < 0.05, **P < 0.01, and ****P < 0.0001, by 1-way ANOVA with Tukey’s multiple-comparison test.

We first examined whether ER stress pathways are activated in mouse ILC3s. We noted that *Ire1α* (also known as *Ern1*) mRNA was highly expressed in ILC3s of the small intestinal lamina propria (siLP) compared with other cell populations in the ImmGen microarray and ULI RNA-Seq data (Immgen.org) (Figure 1A). Previous studies demonstrated that the expression of prototypic ILC3 genes, such as *Il22*, follows a 24-hour circadian rhythm as does expression of clock genes like period circadian regulator 1 (*Per1*) (2, 3, 23). Thus, we tested whether IRE1α/XBP1 expression also follows a cyclic oscillation in ILC3s at steady state. We sort purified siLP ILC3s (Supplemental Figure 1A; supplemental material available online with this article; https://doi.org/10.1172/JCI174198DS1) from WT C57BL/6J mice every 6 hours over a 24-hour period for quantitative real-time PCR (qPCR) analysis of genes controlling the ER stress response. Using JTK_CYCLE analysis, we found robust rhythmic expression of *Xbp1s* peaking at ZT12 over a 24-hour period, which paralleled that of *Il22* and *Per1* (Figure 1B and Supplemental Methods). Circadian oscillations of *Pdi*, which encodes an ER protein disulfide isomerase, and *Bip*, an ER chaperone gene, were limited. We also observed cyclic expression of *Chop* (*Ddit3*, or DNA damage–inducible transcript 3), which encodes a proapoptotic transcription factor during ER stress (11). However, the expression of *Chop* peaked at ZT0 when *Xbp1s*, *Per1*, and *Il22* transcripts were at their nadir, suggesting that CHOP may counterbalance the activity of the IRE1α/XBP1 pathway in intestinal ILC3s. VIP is a neuropeptide produced by enteric neurons during feeding, which was previously shown to induce the rhythmic expression of ILC3 cytokines including IL-22 (24–26). We found that ex vivo treatment of siLP ILC3s with VIP or the VIP receptor agonist BAY 55-9837 upregulated XBP1s in siLP ILC3s (Figure 1C). This finding suggests a strong connection between the nutritional response and the circadian regulation of IRE1α/XBP1. Moreover, it implies that VIP secretion induced by food intake might regulate cytokine production in gut ILC3s via the IRE1α/XBP1 pathway.

### ER stress enhances ILC3 production of cytokines.

**Figure 2:**
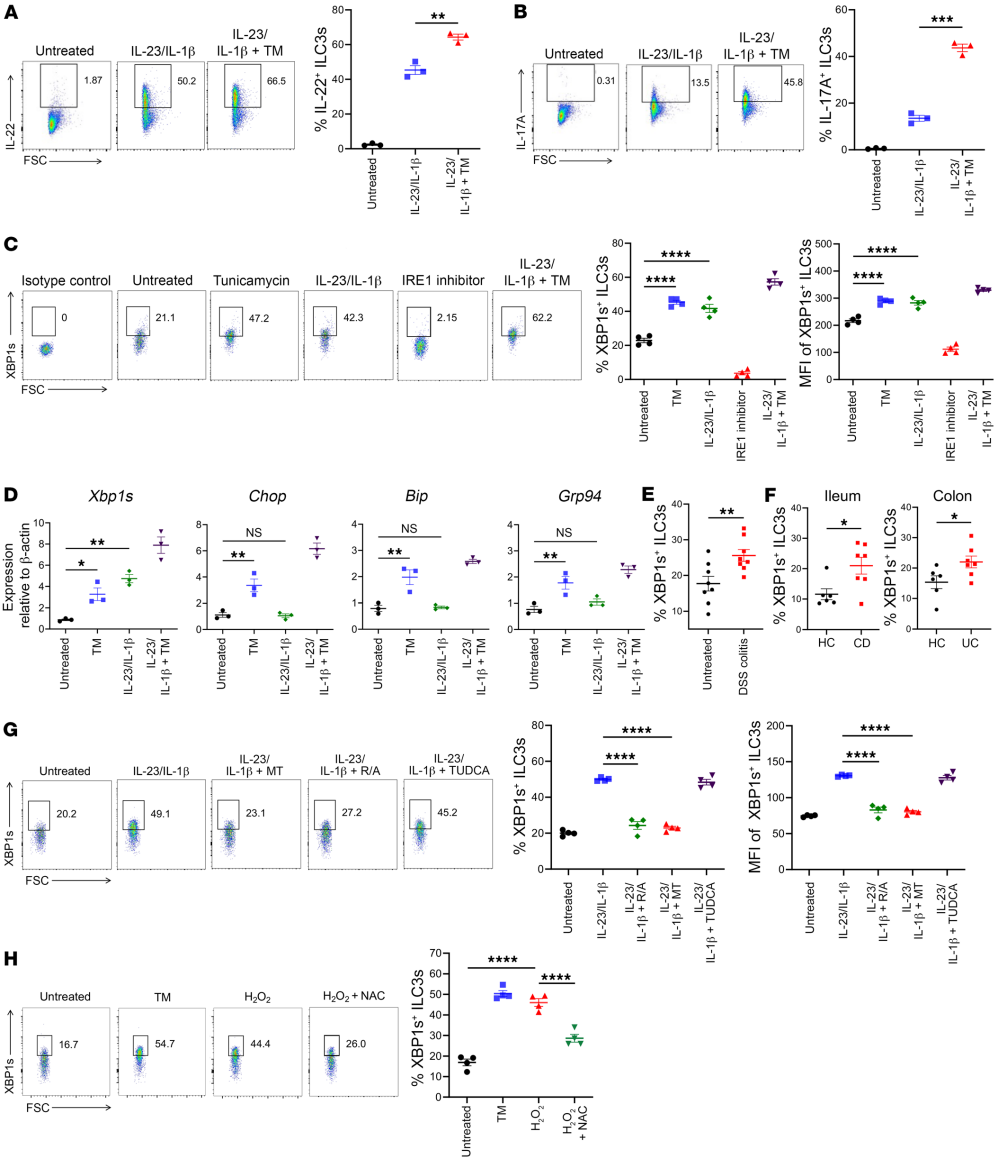
Inflammation and IBD activate IRE1α-XBP1 in ILC3s, which requires mtROS. (**A** and **B**) Sorted mouse siLP ILC3s (all subsets included) were untreated or treated with 1 ng/mL IL-23 combined with 1 ng/mL IL-1β or 5 μg/mL TM, as noted, for 4 hours (GolgiPlug for the last 3 hours), and intracellular cytokines were assessed by flow cytometry. (**C** and **D**) Sorted mouse siLP ILC3s (all subsets included) were treated or not with 1 ng/mL IL-23, 1 ng/mL IL-1β, 5 μg/mL TM, or 10 μM IRE1 inhibitor 4μ8C or their combination, as noted, for 4 hours. (**C**) Intracellular XBP1s was measured by flow cytometry. (**D**) Expression of UPR components was measured by qPCR relative to β-actin (*n* = 3). (**E**) WT C57BL/6J mice were either untreated or given 3% DSS in drinking water for 5 days to induce acute colitis, and colonic LP ILC3s were isolated for flow cytometry. The percentage of XBP1s^+^ colonic LP ILC3s from each mouse is shown (*n* = 8). (**F**) Inflamed ileal (for CD) or colonic (for UC) samples and ileal/colonic tissue from healthy controls (HC) were collected for isolation of lymphocytes. Human ILC3s were identified as live CD45^+^Lin^–^CD127^+^NKp44^+^ lymphocytes. Intracellular XBP1s in ILC3s was detected by flow cytometry. The percentages of XBP1s^+^ ILC3s in samples from HC, CD and UC patients are shown in the panels. (**G**) Sorted mouse siLP ILC3s were untreated or treated as noted with 10 ng/mL IL-23, 1 ng/mL IL-1β, 50 μM MitoTEMPO (MT), 0.1 μM rotenone plus 1 μM antimycin A (R/A), 5 mM TUDCA, or their combination for 6 hours. (**H**) Mouse siLP ILC3s were treated with 5 μg/mL TM or with 20 μM hydrogen peroxide (H_2_O_2_) with or without 5 mM NAC for 4 hours. XBP1s was measured by intracellular staining. Representative plots are shown. All data represent at least 2 independent experiments. Error bars indicate the SEM. *P < 0.05, **P < 0.01, ***P < 0.001, and ****P < 0.0001, by 2-tailed Student *t* test (**E** and **F**) or 1-way ANOVA with Tukey’s multiple-comparison test (**A**–**D**, **G**, and **H**).

To directly investigate whether ER stress affects cytokine production of ILC3s, we sorted siLP ILC3s from WT C57BL/6J mice (Supplemental Figure 1A). ILC3s were stimulated ex vivo with IL-23 plus IL-1β with or without tunicamycin (TM), a classical ER stress inducer that activates all 3 branches of the UPR (i.e., IRE1α, PERK, and ATF6). Stimulation of ILC3s with IL-23 plus IL-1β induced production of IL-22 and IL-17A, which were enhanced by cotreatment with TM (Figure 2, A and B, and Supplemental Figure 1, B and C). Stimulation of ILC3s with TM alone had a minimal effect on the expression of cytokines (Supplemental Figure 1C). We conclude that ER stress enhanced ILC3 production of cytokines induced by proinflammatory cytokines.

### ILC3s exposed to inflammatory cytokines, experimental colitis, and IBD selectively activate IRE1α/XBP1.

We further investigated the expression of the IRE1α/XBP1 pathway during ILC3 activation ex vivo and in vivo. We isolated siLP ILC3s, stimulated them ex vivo with IL-23 plus IL-1β or TM, and examined the intracellular content of XBP1s in ILC3s by flow cytometry. Stimulation of siLP ILC3s with either IL-23 plus IL-1β or TM induced similar expression levels of XBP1s, suggesting that inflammatory cytokines were sufficient to activate the IRE1α/XBP1 branch of the UPR pathway. Moreover, TM, together with IL-23 and IL-1β, had an additive effect (Figure 2C). We further examined the expression of ER stress genes in sorted siLP ILC3s by qPCR. This analysis corroborated that IL-23 and IL-1β selectively activated the IRE1α/XBP1 branch of the UPR but had a limited effect on the other 2 UPR branches — the PERK pathway (*Chop*) and the ATF6 pathway (*Bip* and *Grp94*) (Figure 2D and Supplemental Methods).

We next examined IRE1α/XBP1 activation in colonic ILC3s in the DSS-induced model of colitis. We collected cells from colonic LP of mice on day 5 of DSS treatment and analyzed intracellular XBP1s in ILC3s by flow cytometry. The percentage of ILC3s expressing XBP1s was higher in mice with DSS colitis than in untreated mice (Figure 2E). The expression of XBP1s remained unchanged in other ILC subsets — ILC1, ILC2, and NK cells — in the colon during DSS colitis (Supplemental Figure 2). Furthermore, we examined the frequencies of XBP1s^+^ ILC3s in inflamed intestinal biopsies from patients with CD or ulcerative colitis (UC), as well as normal mucosal tissues from healthy controls (*n* = 6 or 7 per group) (Figure 2F and Supplemental Figure 3). The data indicated that the IRE1α/XBP1 pathway in ILC3s is also activated in response to intestinal inflammation in patients with IBD.

### IRE1α/XBP1 activation in ILC3s requires mtROS.

We sought to elucidate the mechanism underpinning IRE1α/XBP1 activation in ILC3 during inflammation. It has been shown that ROS generated by the mitochondrial electron transport chain (ETC) can induce ER stress (27) and sustain ILC3 activation (28). Thus, we investigated potential involvement of mtROS in XBP1s expression. Sort-purified mouse siLP ILC3s were stimulated with IL-23 and IL-1β with or without mtROS inhibitors, including the mitochondria-targeted antioxidant MitoTEMPO as well as the mitochondrial ETC complex I/III inhibitors rotenone and antimycin A. We also tested the effect of tauroursodeoxycholic acid (TUDCA), a chemical chaperone that alleviates global ER stress by improving ER protein folding (17). Cell lysates were analyzed by qPCR for the expression of *Xbp1s* mRNA. Treatment with either MitoTEMPO or rotenone plus antimycin A thwarted IL-23/IL-1β–induced *Xbp1* splicing in ILC3s. However, the effect of TUDCA on IL-23/IL-1β–induced *Xbp1s* expression was limited (Supplemental Figure 4). These results were confirmed by intracellular staining of XBP1s in ILC3s (Figure 2G and Supplemental Figure 5). Furthermore, we exposed mouse siLP ILC3s to hydrogen peroxide for 4 hours to induce ROS (29), resulting in the upregulation of XBP1s in ILC3s. This induction of XBP1s was reversed by the antioxidant *N*-acetyl cysteine (NAC) (Figure 2H). In contrast, cotreatment with the PERK inhibitor GSK2606414 did not alter the level of XBP1s (30) (Supplemental Figure 5). These data demonstrate that IRE1α/XBP1 activation in stimulated ILC3s required ROS generated by the mitochondrial ETC, a vital event for ILC3 activation (28). It also suggests that the selective IRE1α/XBP1 activation in ILC3s was distinct from the canonical ER stress response, which involves all 3 UPR branches and is attenuated by chemical chaperones such as TUDCA (11).

### IRE1α/XBP1 augments cytokine production in mouse and human ILC3s.

**Figure 3:**
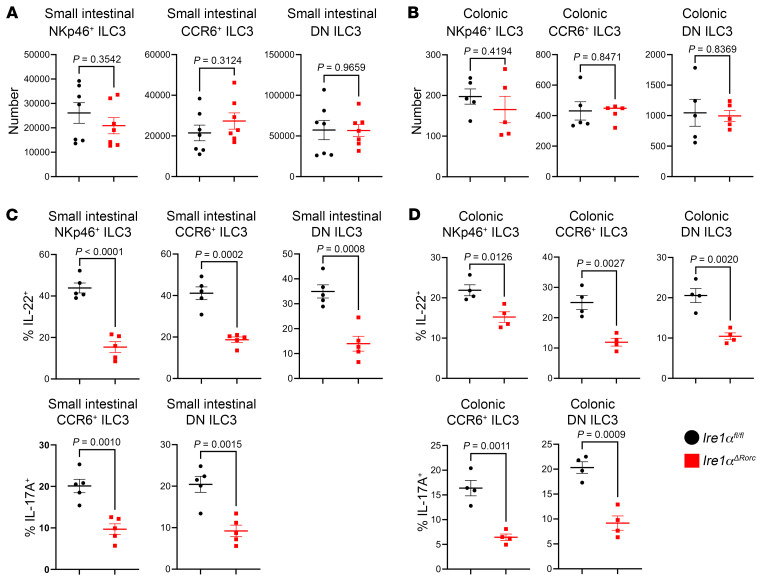
ER stress and IRE1α-XBP1 control cytokine production in ILC3s. Mouse ILC3s were identified as live CD45^int^Lin^–^CD90.2^hi^ lymphocytes, which were NKp46^+^, CCR6^+^, or NKp46^–^CCR6^–^ (DN). ILC3 cell counts in *Ire1α^ΔRorc^* and *Ire1α^fl/fl^* siLP (**A**) and colonic LP (**B**). siLP cells (**C**) and colonic LP cells (**D**) were treated with 1 ng/mL IL-23 and 1 ng/mL IL-1β for 4 hours (GolgiPlug for the last 3 hours) and intracellular IL-17A and IL-22 levels were measured in CCR6^+^, NKp46^+^, and DN ILC3 subsets by flow cytometry (*n* = 4–5). Data represent at least 3 independent experiments each involving 4–7 mice per group. *P* values, shown above the bars in the plots, were calculated using an unpaired, 2-tailed Student’s *t* test. Error bars indicate the SEM.

Given that ER stress sustains ILC3 cytokine production and that inflammatory stimuli selectively activate IRE1α/XBP1 in ILC3s, we next asked whether the IRE1α/XBP1 pathway is required for ILC3 cytokine production. We crossed *Ire1α^fl/fl^* mice with *Rorc-Cre* mice to generate *Ire1α^ΔRorc^* mice. The deletion of exons 16 and 17 of *Ire1α* disrupts its endoribonuclease activity, precluding the formation of *Xbp1s* mRNA (31). All ILC3 subsets (CCR6^+^, NKp46^+^, and double-negative [DN] CCR6^–^NKp46^–^) were equally represented in the small intestine and colon of *Ire1α^ΔRorc^* mice and their *Ire1α^fl/fl^* littermates (Figure 3, A and B). Similarly, the pool of RORγt^+^ T cells in the small intestine and colon of *Ire1α^ΔRorc^* mice were unchanged at steady state (Supplemental Figure 6). However, fewer siLP and colonic ILC3s from *Ire1α^ΔRorc^* mice stimulated ex vivo with IL-23 and IL-1β produced IL-22 and IL-17A than did ILC3s from *Ire1α^fl/fl^* littermates (Figure 3, C and D). These data indicate that the IRE1α/XBP1 pathway directly contributed to cytokine production in response to inflammatory cytokines in all ILC3 subsets.

### Ire1α^ΔRorc^ mice are vulnerable to intestinal infection due to impaired ILC3s.

**Figure 4:**
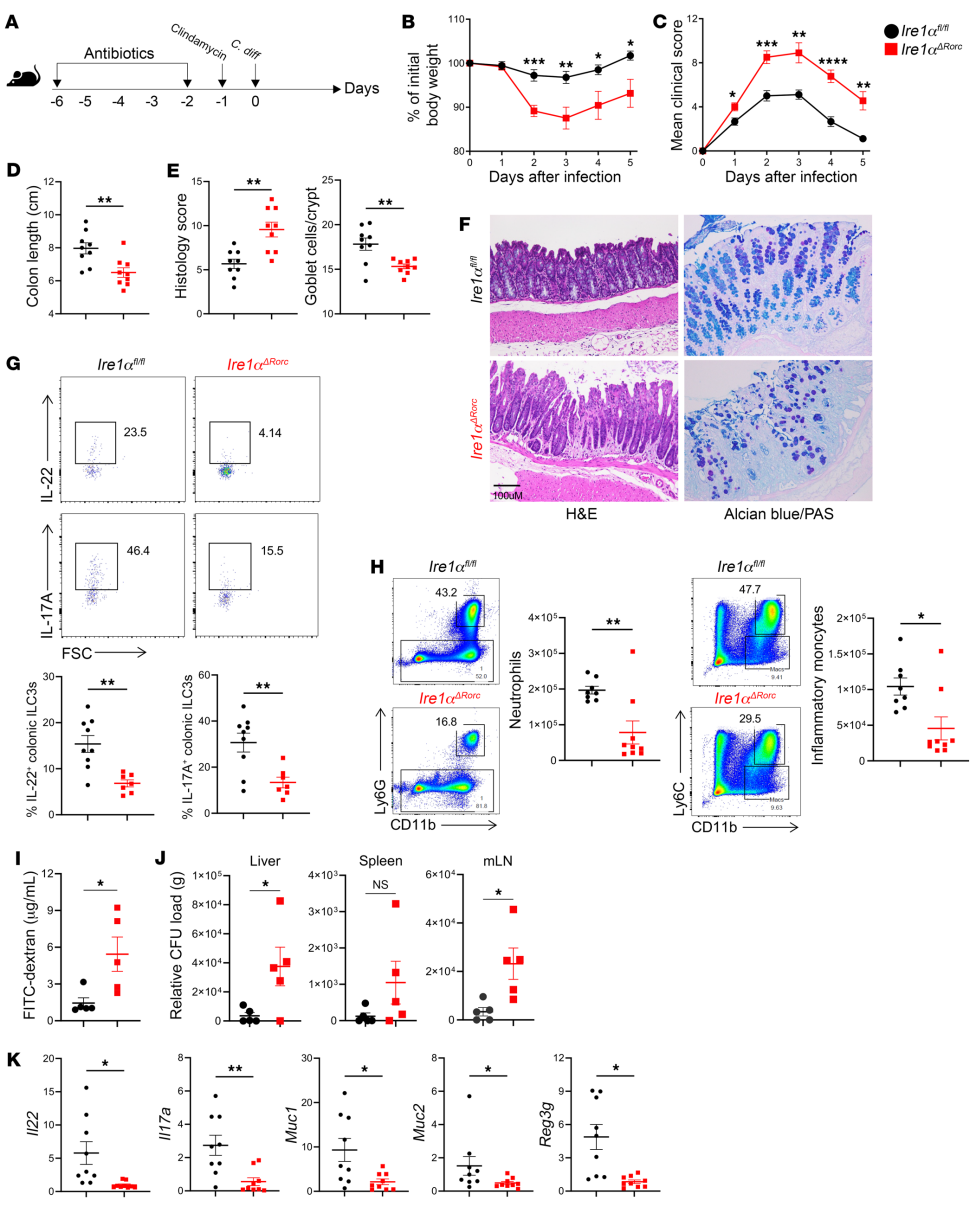
*Ire1α^ΔRorc^* mice are highly susceptible to *C*. *difficile* infection. (**A**) *Ire1α^ΔRorc^* and *Ire1α^fl/fl^* mice were orally infected by *C*. *difficile* (*C. diff*) following treatment with antibiotics. (**B**) Weight loss and (**C**) clinical scores were measured daily (*n* = 9–10). (**D**–**F**) Mice were sacrificed on day 5 after infection and colons harvested for measurement of length (**D**), as well as histology score and goblet cell numbers (**E**) based on H&E staining and alcian blue/PAS staining (**F**). Scale bar: 100 μm. (**G**) Colonic LP ILC3s were isolated on day 5 after infection and stimulated ex vivo with 10 ng/mL IL-23 and 10 ng/mL IL-1β for 4 hours (GolgiPlug for the last 3 hours); intracellular cytokine levels were measured by flow cytometry (*n* = 7–9). Representative plots are on the top, and the percentage of cytokine^+^ ILC3s in each are mouse shown on the bottom. (**H**) Representative plots and absolute number of neutrophils and inflammatory monocytes in the colonic LP of *C*. *difficile*–infected mice on day 2 after infection (*n* = 8–9). (**I**) Mice received FITC-dextran by gavage on day 3 after infection, and serum was collected 4 hours later and FITC-dextran quantitated (*n* = 5). (**J**) Bacterial translocation into the liver, spleen, and mLNs was assessed by qPCR on day 3 after infection (*n* = 5). (**K**) Expression of cytokines, mucins, and antimicrobial peptides in the proximal colon on day 5 after infection was measured by qPCR relative to β-actin (*n* = 7–9). Data represent at least 2 independent experiments, each involving 5–9 mice per group. Error bars indicate the SEM. *P < 0.05, **P < 0.01, ***P < 0.001, and ****P < 0.0001, by unpaired, 2-tailed Student’s *t* test.

Since fewer ILC3s from *Ire1α^ΔRorc^* mice produced protective cytokines, we assessed the functional relevance of IRE1α/XBP1 deficiency in ILC3s in models of intestinal infection that cause barrier damage and activate ILC3s. We chose to examine *Ire1α^ΔRorc^* mice in the acute phases of infection by either *Clostridium difficile* or *Citrobacter rodentium*, since ILC3-derived IL-22 is essential to preserve epithelial barrier function, while CD4^+^ T cells are dispensable at this stage (28, 32–35). The small intestine and colon of *Ire1α^ΔRorc^* mice and control littermates appeared morphologically similar in the absence of infection (Supplemental Figure 7). Mice were orally administrated *C*. *difficile* after treatment with antibiotics to induce dysbiosis (Figure 4A). *Ire1α^ΔRorc^* mice lost more weight and had higher clinical scores than did their *Ire1α^fl/fl^* littermates (Figure 4, B and C). Consistently, *Ire1α^ΔRorc^* mice had more severe colitis, as indicated by shorter colon lengths, histology scores, as well as goblet cell loss visualized by alcian blue/PAS staining (Figure 4, D–F). On day 5 after infection, fewer colonic ILC3s from *Ire1α^ΔRorc^* mice produced IL-22 and IL-17A than did ILC3s from *Ire1α^fl/fl^* mice (Figure 4G). ILC3 cytokines are essential for recruitment of innate immune cells including neutrophils during acute *C*. *difficile* infection (33). In comparison with *Ire1α^fl/fl^* mice, recruitment of neutrophils and inflammatory monocytes was impaired in the colon of *Ire1α^ΔRorc^* mice (Figure 4H and Supplemental Figure 8), as was compromised intestinal barrier integrity gauged by FITC-dextran permeability (Figure 4I), and exacerbated bacterial translocation to the liver and mesenteric lymph nodes (mLNs) (Figure 4J). Moreover, expression of mucins and antimicrobial peptides was diminished in the colonic tissue of *Ire1α^ΔRorc^* mice with *C*. *difficile* infection (Figure 4K and Supplemental Methods). Since *Rorc*-*Cre* is expressed not only in ILC3s but also in T lymphocytes, we examined *Ire1α^ΔRorc^* *Rag1^–/–^* and *Ire1α^fl/fl^* *Rag1^–/–^* control mice to account for any potential effects on T cells. Upon acute *C*. *difficile* infection, *Ire1α^ΔRorc^* *Rag1^–/–^* mice developed more severe disease, as indicated by weight loss, clinical scores, shortening of colon length, histology score, and goblet cell depletion (Supplemental Figure 9), recapitulating the phenotype of *Ire1α^ΔRorc^* mice. In addition, *Ire1α^ΔRorc^* mice were also more susceptible than *Ire1α^fl/fl^* mice to *C*. *rodentium* infection, as assessed by their progressive weight loss, CFU in the feces, shortening of colon length, histology score, goblet cell loss (alcian blue/PAS), and epithelial cell proliferation (Ki67) (Supplemental Figure 10) (36–38). Overall, these data demonstrated that impaired production of ILC3-derived cytokines rendered *Ire1α^ΔRorc^* mice highly susceptible to acute infection by *C*. *difficile* or *C*. *rodentium*.

### Ire1α^ΔRorc^ mice are more susceptible to DSS-induced colitis.

**Figure 5:**
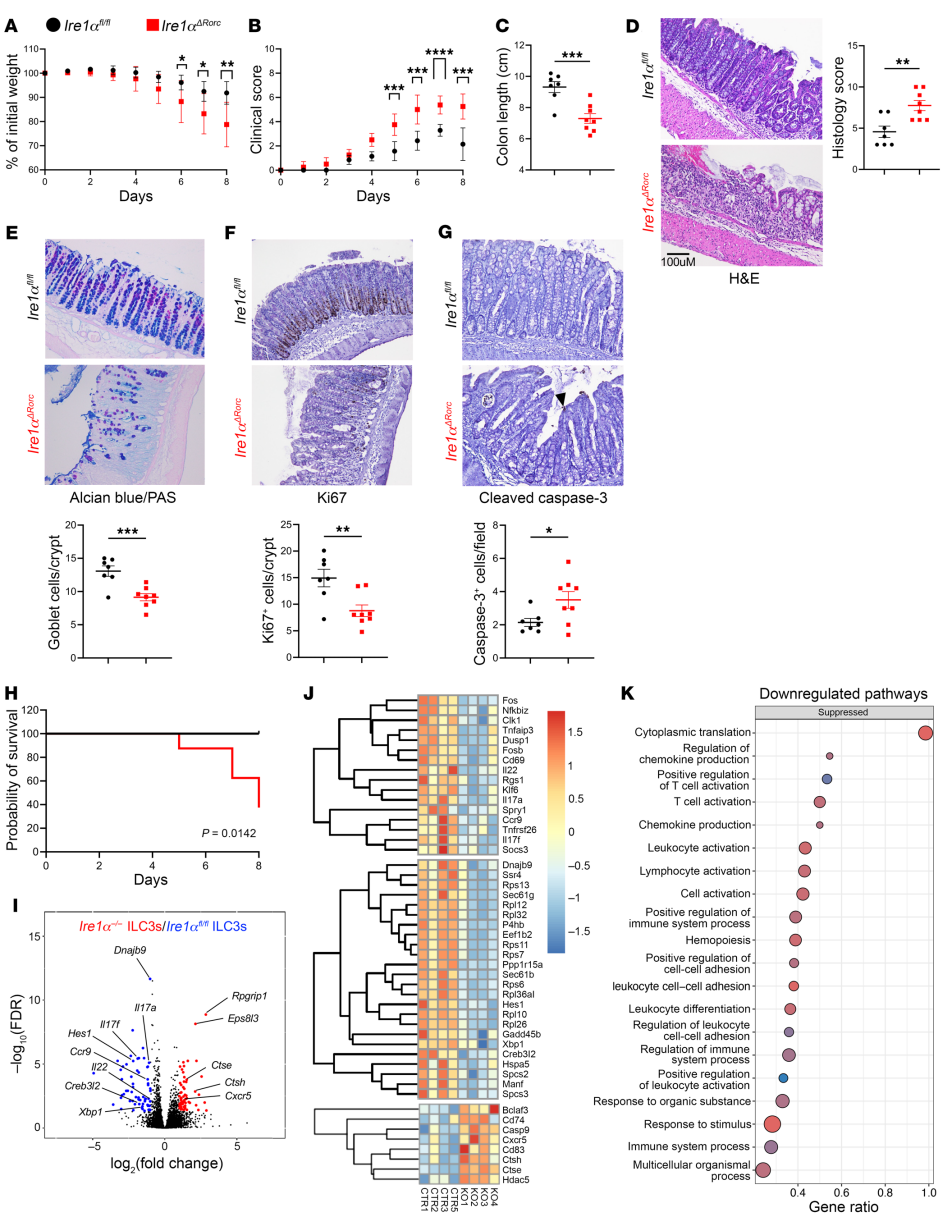
*Ire1α^ΔRorc^* mice are more susceptible to DSS-induced colitis. (**A**–**E**) *Ire1α^ΔRorc^* and *Ire1xα^fl/fl^* mice were given 3.5% DSS in drinking water for 7 days followed by 1 day of fresh, untreated water. (**A**) Weight loss and (**B**) clinical scores were measured daily (*n* = 7 or 8). Error bars indicate SD in **A** and **B**. Mice were sacrificed on day 8 of colitis, and colons were harvested. The following parameters were measured: colon length (**C**); histology score determined by H&E staining (**D**); goblet cell numbers assessed via alcian blue/periodic acid–Schiff (PAS) staining (**E**); and proliferating and apoptotic cells identified through IHC staining for Ki67 (**F**) and cleaved caspase-3 (**G**), respectively. Scale bar: 100 μm (**D**–**G**). (**H**) *Ire1α^ΔRorc^* and *Ire1α^fl/fl^* mice were given 4% DSS in drinking water for 8 days and mortality was assessed (*n* = 7 or 8). Data represent at least 2 independent experiments. (**I**–**K**) Bulk RNA-Seq of colonic ILC3s from *Ire1α^ΔRorc^* and *Ire1α^fl/fl^* mice with DSS colitis (*n* = 4). (**I**) Volcano plot of genes with greater than 1.5-fold differential expression in *Ire1α^fl/fl^* versus *Ire1α^ΔRorc^* ILC3s (blue) and greater than 1.5-fold differential expression in *Ire1α^ΔRorc^* versus *Ire1α^fl/fl^* ILC3s (red). (**J**) Heatmap shows selected target genes that were differentially expressed in *Ire1α^ΔRorc^* ILC3s. (**K**) Downregulated pathways in *Ire1α^ΔRorc^* ILC3s by GO enrichment analysis. Error bars indicate the SEM. *P < 0.05, **P < 0.01, ***P < 0.001, and ****P < 0.0001, by 2-tailed Student’s *t* test.

To further assess the effect of IRE1α/XBP1 on ILC3 functions in vivo, we examined DSS-induced acute colitis, an epithelial injury model of IBD. *Ire1α^ΔRorc^* mice lost more weight and had higher clinical scores than did *Ire1α^fl/fl^* controls upon DSS challenge (Figure 5, A and B). Additionally, *Ire1α^ΔRorc^* mice had shorter colons and higher histology scores with acute colitis (Figure 5, C and D). *Ire1α^ΔRorc^* mice with colitis also had extensive goblet cell depletion, reduced epithelial cell proliferation, and elevated epithelial cell apoptosis, as visualized by staining with alcian blue/PAS, Ki67, and cleaved caspase-3, respectively (Figure 5, E–G). When challenged with a higher dose of DSS in the drinking water, fewer *Ire1α^ΔRorc^* mice than control littermate mice survived (Figure 5H). These data demonstrate that *Ire1α^ΔRorc^* mice were more susceptible to DSS-induced acute colitis. To dissect the potential confounding effect of T cells in the observed phenotype, we repeated the experiment using *Ire1α^ΔRorc^* *Rag1^–/–^* and *Ire1α^fl/fl^* *Rag1^–/–^* control mice. *Ire1α^ΔRorc^* *Rag1^–/–^* mice developed more severe acute DSS colitis, as indicated by weight loss, clinical score, shortening of colon length, histology score, goblet cell loss (alcian blue/PAS), and epithelial cell proliferation (Ki67) (Supplemental Figure 11), corroborating the phenotype of the *Ire1α^ΔRorc^* mice.

To investigate the mechanisms underpinning the susceptibility of *Ire1α^ΔRorc^* mice to DSS-induced colitis, we sort-purified colonic ILC3s from *Ire1α^ΔRorc^* and *Ire1α^fl/fl^* mice with DSS colitis for bulk RNA-Seq. In comparison with control ILC3s, skewed expression of a broad spectrum of genes was evident in *Ire1α^ΔRorc^* ILC3s (Figure 5, I and J, and Supplemental Figure 12). The downregulated transcripts in *Ire1α^ΔRorc^* ILC3s included ER stress response/UPR-associated genes, such as those encoding ER chaperones (*Dnajb9*, *Hspa5*), transcription factors (*Nfkbiz*, *Hes1*, *Creb3I2*), and a neurotrophic factor (*Manf*). Expression of mRNAs encoding ribosomal proteins (*Rpl*, *Rps*), signal peptidase complex subunits (*Spcs2*, *Spcs3*), Sec61 translocon components (*Sec61b*, *Sec61g*, *Ssr4*), which control mRNA translation and ER protein translocation, also declined in *Ire1α^ΔRorc^* ILC3s. In addition, transcripts for cytokines, including *Il22*, *Il17a*, *Il17f*, and several activation markers, such as *Ccr9*, *Cd69*, *Fos*, and *Dusp1*, were diminished in *Ire1α^ΔRorc^* ILC3s. Genes showing heightened expression in *Ire1α^ΔRorc^* ILC3s included *Cxcr5*, which suppresses cytokine secretion of CCR6^+^ ILC3s (39), various components of the antigen presentation machinery (*Ctse*, *Ctsh*, *Cd74*, *Cd83*), and several genes involved in proapoptotic signaling (*Bclaf3*, *Casp9*). Gene ontology (GO) enrichment analysis uncovered decreased pathways in *Ire1α^ΔRorc^* ILC3s such as cytoplasmic translation, chemokine production, and lymphocyte activation (Figure 5K and Supplemental Figure 13). In conclusion, transcriptomic analysis of colonic ILC3s from *Ire1α^ΔRorc^* mice with DSS-induced colitis corroborated the diminished expression of ER stress genes and cytokines and further indicated the induction of proapoptotic genes that can contribute to dysregulated ILC3 functions.

### Exogenous IL-22 accelerates recovery from acute DSS colitis in Ire1α^ΔRorc^ mice.

**Figure 6:**
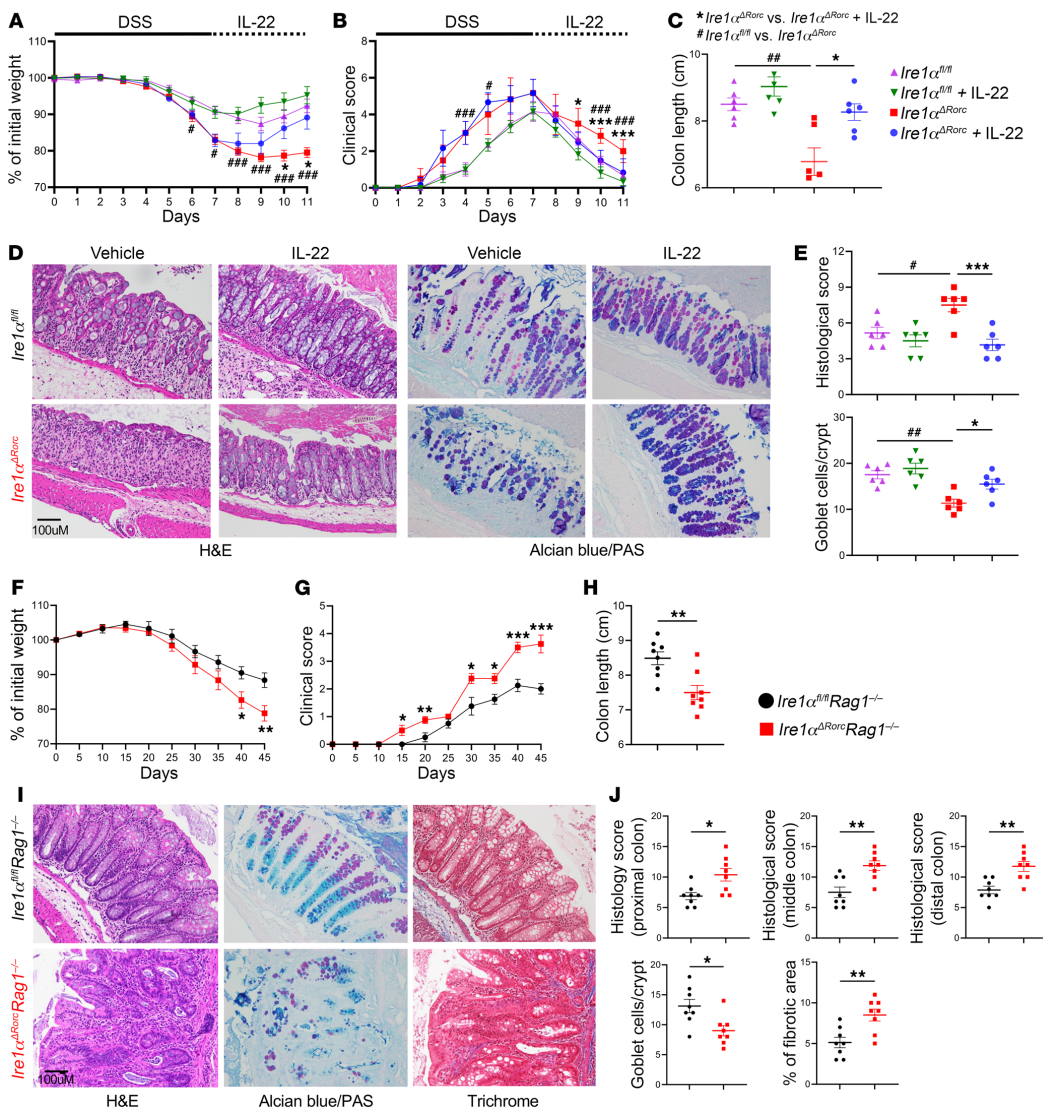
Loss of *Ire1α* in ILC3s impedes recovery from acute DSS colitis and exacerbates T cell transfer–induced colitis. *Ire1α^ΔRorc^* mice were given 3.5% DSS in drinking water for 7 days to induce acute colitis, followed by i.p. injection of 1 μg mouse recombinant IL-22 daily on days 7–11. (**A**) Weight loss and (**B**) clinical scores were measured daily. Mice were sacrificed on day 11, and colons were harvested. The following parameters were measured: colon length (**C**); histology score determined by H&E staining (**D** and **E**); and goblet cell numbers assessed by alcian blue/PAS staining (**D** and **E**) (*n* = 6). Scale bar: 100 μm. (**F**–**J**) Adoptive transfer of WT CD4^+^CD45RB^hi^ T cells into *Ire1α^ΔRorc^* *Rag1^–/–^* and *Ire1α^fl/fl^* *Rag1^–/–^* (control) littermates to induce chronic colitis. (**F**) Weight loss and (**G**) clinical scores were measured every 5 days. Mice were sacrificed on day 45 after transfer, and colons were harvested for measurement of colon length (**H**) and H&E staining, alcian blue/PAS staining, and Masson’s trichrome staining (**I** and **J**) (*n* = 8). Scale bar: 100 μm. Data represent 2 independent experiments. Error bars indicate the SEM. *P < 0.05 or ^#^*P* < 0.05, **P < 0.01 or ^##^*P* < 0.01, and ***P < 0.001 or ^###^*P* < 0.001, by 2-tailed Student *t* test (**F**–**H** and **J**) or 1-way ANOVA with Tukey’s multiple-comparison test (**A**–**C** and **E**).

Given the diminished production of IL-22 by *Ire1α^ΔRorc^* ILC3s, we next explored whether exogenous IL-22 could mitigate the susceptibility of *Ire1α^ΔRorc^* mice to DSS-induced colitis. *Ire1α^ΔRorc^* and *Ire1α^fl/fl^* mice were subjected to a 7-day regimen of 3.5% DSS to induce acute colitis, followed by a recovery period from days 7–11, without DSS treatment. During this recovery phase, 1 group of mice received daily i.p. injections of mouse recombinant IL-22, while another group received vehicle only. The *Ire1α^ΔRorc^* mice exhibited less recovery compared with *Ire1α^fl/fl^* mice. However, treatment with IL-22 significantly improved recovery in *Ire1α^ΔRorc^* mice, as evidenced by changes in weight, clinical scores, colon length, histology scores, and goblet cell numbers (Figure 6, A–E). In contrast, administration of IL-22 had less effect on the recovery of *Ire1α^fl/fl^* mice with normal ILC3s. These findings highlight the critical role of IL-22 deficiency in the heightened susceptibility of *Ire1α^ΔRorc^* mice to intestinal inflammation.

### Ire1α^ΔRorc^ Rag1^–/–^ mice develop exacerbated colitis upon adoptive T cell transfer.

To explore the involvement of IRE1α/XBP1 in intestinal ILC3s beyond acute colitis models, we conducted a T cell transfer–induced colitis experiment, which represents a chronic immune-mediated model of IBD (40, 41). CD4^+^CD45RB^hi^ T cells were sort-purified from WT C57BL/6 mice and transferred into *Ire1α^ΔRorc^* *Rag1^–/–^* and *Ire1α^fl/fl^* *Rag1^–/–^* (control) littermates. After adoptive transfer, *Ire1α^ΔRorc^* *Rag1^–/–^* mice developed more severe colitis, as indicated by progressive weight loss, clinical scores, colon length, and histological changes up to day 45 after transfer (Figure 6, F–J). In addition to inflammation, *Ire1α^ΔRorc^* *Rag1^–/–^* mice showed more severe goblet cell depletion and fibrosis by alcian blue/PAS and Masson’s trichrome staining, respectively (Figure 6, I and J). These data indicate that IRE1α/XBP1 in ILC3s is protective against chronic, immunological colitis in mice, which further supports its clinical relevance in human IBD.

### IRE1 modulators control the production of IL-22 in human intestinal ILC3s.

**Figure 7:**
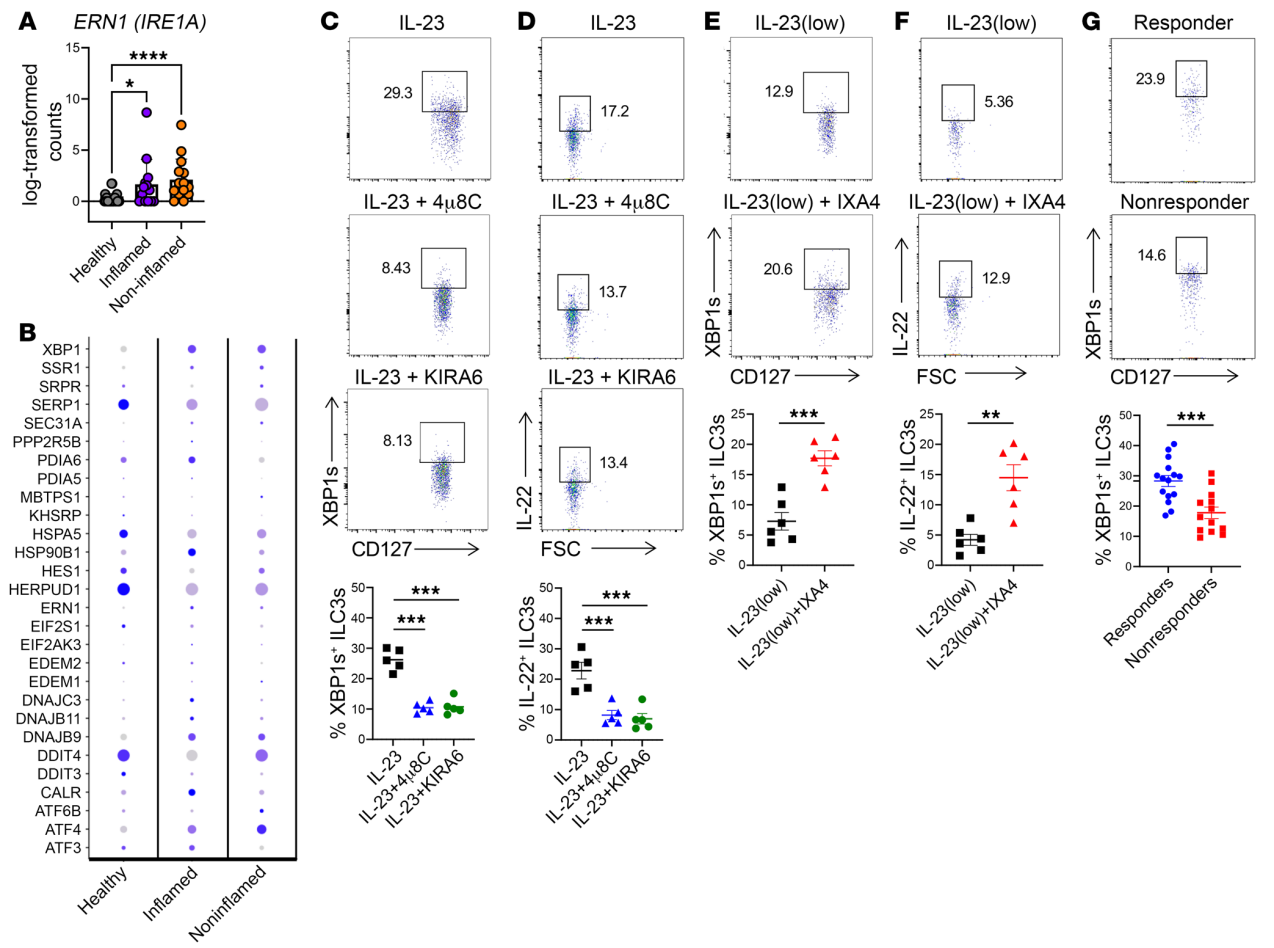
IRE1 modulators orchestrate cytokine production in human ILC3s; intestinal XBP1^+^ ILC3s positively correlate with response to ustekinumab in patients with CD. (**A**) Expression of *ERN1* (*IRE1A*) in colonic ILC3s from healthy controls and from inflamed and noninflamed tissues of patients with UC. (**B**) Expression of UPR genes in colonic ILC3s from healthy controls and from inflamed and noninflamed tissues of patients with UC. (**C**–**F**) ILC3s were sort-purified from colonic biopsies collected from healthy individuals, treated as noted with IL-23 (10 ng/mL in **C** and **D**; 0.1 ng/mL in **E** and **F**), 10 μM 4μ8C, 1 μM KIRA6, or 10 μM IXA4 for 10 hours (with GolgiPlug for the last 4 hours). Intracellular XBP1s (**C** and **E**) and IL-22 (**D** and **F**) levels were measured by flow cytometry (*n* = 5–6). Representative plots and the percentage of IL-22^+^ ILC3s in each sample are shown. (**G**) ILC3s were isolated from inflamed mucosal biopsies collected from patients with CD before starting ustekinumab. ILC3 markers and intracellular XBP1s were detected by flow cytometry. Representative plots are shown on the top, and the percentage of XBP1s^+^ ILC3s in each sample shown on the bottom (total *n* = 28). Error bars indicate the SEM. *P < 0.05, **P < 0.01, ***P < 0.001, and ****P < 0.0001, by 2-tailed Student *t* test (**E**–**G**) or 1-way ANOVA with Tukey’s multiple-comparison test (**A**, **C**, and **D**).

Considering the crucial function of IRE1α in mouse ILC3s, we examined the expression of IRE1α (encoded by *ERN1* in human) and other ER stress genes in human intestinal ILC3s during IBD. We reanalyzed the scRNA-Seq data on colonic LP cells from healthy individuals and patients with UC in a published database (42). Colonic ILC3s from both inflamed and noninflamed UC mucosa showed increased expression of *ERN1* compared with those from healthy controls (Figure 7A). Additionally, expression of *ATF4*, *SSR1*, and *DNAJB9*, which are transactivated by XBP1s (43–45), was elevated in both inflamed and noninflamed UC tissues. In contrast, expression of *PDIA6*, *HSPA5*, *HERPUD1*, and *DDIT4* showed minimal changes or was downregulated in UC tissues (Figure 7B). These findings are consistent with our observation that inflammation specifically activates the IRE1α/XBP1 branch of the UPR in ILC3s.

Since IRE1α/XBP1 enhances mouse ILC3 activation, we next asked whether this pathway also affects the function of human intestinal ILC3s. We recruited adult patients without a history of digestive disease who were undergoing colonoscopy for colon cancer screening. Colonic biopsies were collected, and ILC3s were FACS sorted and stimulated ex vivo with IL-23, with or without IRE1 modulators; these included the selective IRE1 inhibitors 4μ8C and KIRA6 (46, 47) and the selective IRE1 activator IXA4 (48). Cotreatment with 10 ng/mL IL-23 and either 4μ8C or KIRA6 reduced the frequencies of XBP1s^+^ and IL-22–producing ILC3s by more than 60% (Figure 7, C and D). Conversely, IXA4 enhanced the frequencies of XBP1s^+^ and IL-22–producing ILC3s induced by a low concentration of IL-23 (0.1 ng/mL) (Figure 7, E and F). We conclude that IRE1α/XBP1 positively regulates the production of IL-22 in human intestinal ILC3s.

### Intestinal XBP1s^+^ ILC3s in patients with CD predict the response to ustekinumab.

Given that the IRE1α/XBP1 pathway augments cytokine production in response to IL-23 by human ILC3s and that IL-23 blockers have been proven effective in CD, we investigated whether the level of pretreatment XBP1s in ILC3s correlates with the response of patients with CD to the nonselective anti–IL-23 antibody ustekinumab. We recruited patients with active CD, which was defined as a simple endoscopic score for CD (SES-CD) segment score of 3 or higher. Biopsies were collected from inflamed intestinal mucosa (*n* = 28) within 3 months before the patients started ustekinumab and cryopreserved for batch processing as we previously described (49). Patients’ responses were assessed clinically and/or endoscopically 8–40 weeks after the initiation of ustekinumab. Elevated frequencies of XBP1s^+^ ILC3s in intestinal mucosa positively correlated with the response to ustekinumab (Figure 7G). These data suggest that the activity of pretreatment IRE1α/XBP1 in intestinal ILC3s predicts the response to ustekinumab.

## Discussion

This study reveals that inflammation in the gastrointestinal tract triggers the IRE1α/XBP1 pathway in ILC3s. In mice, this pathway was crucial for optimal production of IL-22 and IL-17 by ILC3s, leading to resistance against acute infections caused by *C*. *difficile* or *C*. *rodentium*, acute colitis induced by DSS, and chronic colitis induced by T cell transfer. In humans, activation of IRE1α/XBP1 amplified IL-22 production by intestinal ILC3s in response to IL-23. A specific activator of IRE1 enhanced IL-22 production by colonic ILC3s when exposed to low concentrations of IL-23, whereas inhibitors of IRE1 dampened IL-22 production in response to IL-23. The ER stress response triggered by inflammation in ILC3s exhibited a noncanonical pattern. Specifically, it selectively engaged the IRE1α/XBP1 pathway without activating the other 2 UPR branches (Supplemental Figure 14). Consequently, TUDCA, a chemical chaperone known to suppress canonical ER stress in various cell types and organs (17, 27, 50), did not significantly affect XBP1s induced by inflammatory cytokines. Furthermore, this response relied on mtROS. As evidence, a brief exposure to low-dose H_2_O_2_ directly stimulated IRE1α/XBP1, while mitochondria-targeted antioxidants and ETC inhibitors decreased XBP1s expression in ILC3s. A previous study showed that ROS enhance ILC3 cytokine production and their ability to defend against *C*. *rodentium* infection (28). Our latest findings further this research, suggesting that activation of IRE1α/XBP1 is at least one of the mechanisms through which ROS stimulate ILC3s. The selective activation of IRE1α/XBP1 by ROS in ILC3s is not unprecedented; in fact, ROS-induced XBP1 activation was also observed in macrophages stimulated through TLRs (51). Whether this pathway is generalizable to other immune cell types is currently unknown.

It is worth noting that ILC3s displayed strong rhythmic expression of *Xbp1s*, reaching its peak at ZT12 and aligning with the expression patterns of *Il22* and the clock gene *Per1*. This finding supports the idea that ILC3 functions, similar to many other functions of the gastrointestinal system, are synchronized with the circadian rhythm. Circadian oscillation of the IRE1α/XBP1 pathway was also observed in the liver and shown to modulate lipid metabolism (52). Enteric neurons are localized adjacent to ILC3s in the gut and secrete neuropeptide VIP upon feeding. VIP induces rhythmic expression of IL-22 via VIPR2 on intestinal ILC3s, although the intracellular pathway downstream of VIPR2 remains unclear (24–26). We discovered that a brief exposure to VIP or a VIPR2 agonist prompted the activation of XBP1s in ILC3s. This suggests that the IRE1α/XBP1 pathway may partly underlie VIP’s regulation of rhythmic ILC3 function in the gut. Interestingly, the expression of *Chop* peaked at ZT0 when *Xbp1s*, *Il22*, and *Per1* transcripts were at their nadir. CHOP is transcription factor mainly activated by the PERK-eIF2α branch of the UPR (11). It has a proapoptotic function during prolonged ER stress but can also repress T-bet (53), which promotes the conversion of IL-22–producing ILC3s into IFN-γ–producing ILC1s in mice (54, 55). Future studies are needed to explore whether CHOP regulates the rhythmicity and plasticity of ILC3s.

Here, we demonstrate that IRE1α/XBP1 strengthens IL-23 signaling in ILC3s, enhancing protection against both acute and chronic colitis in mice. This finding is somewhat unexpected, given that dysregulated IL-23 is known to be pathogenic in human IBD. One potential explanation for this apparent paradox is that IRE1α/XBP1-enhanced IL-23 signaling may be beneficial in ILC3s by stimulating the expression of IL-22, which improves epithelial integrity and impedes bacterial translocation. Conversely, it may be detrimental in other cell types, including Th17, Th1-like, IL-17–expressing cytotoxic T cells (Tc17), γδ T cells, and NKT cells, where IL-23 promotes the production of proinflammatory cytokines such as TNF-α, IFN-γ, and IL-6 (56) (Supplemental Figure 14). Another potential explanation is that IL-23 activates multiple downstream pathways in addition to IRE1α/XBP1, such as the JAK/STAT pathways, which are important drug targets in IBD. While the IL-23-IRE1α/XBP1 pathway in ILC3s may be protective in colitis, other IL-23 targets including JAK2 and TYK2 are pathogenic (57). This may explain why the loss of IRE1α/XBP1 in ILC3s exacerbates colitis in mice, while global suppression of IL-23 benefits a subset of patients with IBD.

Therapies targeting IL-23, such as ustekinumab and risankizumab, have emerged as crucial strategies in managing IBD. However, over half of the patients with IBD do not respond to IL-23 antagonists, and currently available biomarkers fail to predict their responses. Notably, levels of IL-23 in peripheral blood or intestinal mucosa have proven ineffective in predicting the response to anti–IL-23 therapies. Our research uncovered that elevated frequencies of pretreatment XBP1s^+^ ILC3s were associated with a more favorable response to ustekinumab. This suggests that XBP1s^+^ ILC3s may indicate increased sensitivity to IL-23 in patients with IBD, making them suitable candidates for biological therapies that diminish IL-23 availability. This finding aligns with previous observations showing that CD patients with higher baseline levels of IL-22 were more likely to benefit from brazikumab, another IL-23 antagonist (58). Another explanation could be that high levels of XBP1s^+^ ILC3s and IL-22 reflect an enhanced capacity of patients to maintain ILC3-mediated protective mechanisms alongside the inhibition of pathogenic T cells (Supplemental Figure 14). Ongoing studies aim to validate the predictive value of XBP1s^+^ ILC3s in larger, independent cohorts of patients with IBD starting ustekinumab or newer selective anti–IL-23 antibodies including risankizumab and mirikizumab.

## Methods

### Sex as a biological variant.

We included both male and female sexes in the mouse and human studies. Sex was not considered as a biological variable in the studies.

### Mice.

*Ire1α^fl/fl^* mice were a gift from Randal Kaufman (University of Michigan Medical Center, Ann Arbor, Michigan, USA) (31). *Rorc-Cre* mice were generated by Gerard Eberle and co-authors (59) and provided by A. Tumanov (University of Texas Health Science Center at San Antonio, San Antonio, Texas, USA). *Rag1^–/–^* mice were purchased from The Jackson Laboratory. All mice used for experiments were on a C57BL/6 background and 8–12 weeks of age at the time of the experiments. Mice were housed in regular filter-top cages with free access to sterile water and food in a pathogen-free barrier facility fully staffed and equipped by the Washington University Division of Comparative Medicine, with daily monitoring by highly trained staff. Environmental conditions were reproducibly regulated for temperature, humidity, lighting, caging, bedding, and water. The room housing the animals was maintained at 30%–70% humidity and temperatures of 20°C–26°C (68°F–79°F) with ventilation sufficient to maintain appropriate temperature and humidity ranges and to control odor. A 12-hour light/12-hour dark cycle was maintained in the animal facility. Practices were followed for consistent husbandry and animal line maintenance per IACUC protocols. For example, we used genotypes from the same litter to control for litter effects. We used single pairs for continuous breeding and refreshed breeders at approximately 8 months for a female and approximately 1 year for a male. To avoid crowding, we kept 5 post-weaned mice per cage, 3 adults and 1 litter only up to 14 days old, or 2 dams with 2 young litters only less than 7 days of age, per the protocol. To avoid phenotypic variability, cohoused sex- and age- matched littermates were randomly assigned to the treatment and control groups. Each treatment group was compared with a control group studied in parallel. Eight-week-old WT C57BL/6 mice for the circadian rhythm study were purchased from The Jackson Laboratory and housed in light-controlled cabinets as previously described (2). We implemented several crucial strategies to mitigate variability in gut microbiome composition within our colitis models (33, 60, 61). First, we adopted the practice of cohorting mice from birth to minimize cage effects, and we ensured the randomization of littermate mice across experimental groups to ensure an equitable distribution of potential sources of variability. As mentioned above, we maintained standardized housing conditions, including elements such as temperature, humidity, and light cycles, to establish a stable and highly controlled environment. Furthermore, we provided a uniform diet for all mice to control dietary influences on the gut microbiome, thereby minimizing fluctuations in microbial composition. These comprehensive measures have collectively contributed to the creation of a more controlled experimental framework in our colitis research.

### Mouse tissue preparation and single-cell isolation.

Small intestinal and colonic LP cells were isolated as we previously described (2, 33, 62). Briefly, mouse intestines were dissected out and washed with ice-cold HBSS/HEPES. Mesenteric fat and Peyer’s patches were removed, and intestines were cut open longitudinally. Intestines were incubated in HBSS-EDTA to remove intestinal epithelium. Intestines were washed, cut into small pieces, and shaken in complete RPMI with collagenase IV at 37°C for digestion (30 minutes for small intestine, 45 minutes for colon). The digestant was then filtered through a 100 μm strainer and washed with ice-cold HBSS/HEPES. LP cells were further purified through a 40/70 Percoll density gradient.

### Patient recruitment.

Adult patients with active CD (SES-CD segment score ≥3) who were about to start ustekinumab were recruited for the study. Mucosal samples were collected within 3 months of the initiation of ustekinumab. Six endoscopic biopsies were taken from inflamed mucosa during routine colonoscopy. Specimens from inflamed intestinal mucosa were identified intraoperatively by the endoscopist and subsequently confirmed by a blinded gastrointestinal pathologist. Samples were cryopreserved within 20–30 minutes of collection as we described previously (49). Briefly, the biopsies were kept in complete RPMI medium on ice immediately after collection and transported to the laboratory, where they were changed into a freezing medium (10% dimethyl sulfoxide in FCS), transferred into a prechilled Mr. Frosty (Thermo Fisher Scientific) container with isopropanol, and then frozen and kept at –80°C. The samples were then transferred to liquid nitrogen 24 hours later for storage.

Patients’ responses were assessed clinically and/or endoscopically 8–40 weeks after the initiation of treatment. Clinical response was defined as a decrease in the Harvey-Bradshaw index (HBI) of 3 or higher, a decrease in the CD activity index (CDAI) of 100 points or more from baseline, or a CDAI below 150. The endoscopic response was defined as a 50% or greater reduction in SES-CD.

### Preparation of single-cell suspensions from endoscopic biopsies.

Frozen specimens were processed in batches to minimize variation as we described previously (49). Briefly, samples were thawed in a 37°C water bath, rinsed with ice-cold PBS, and digested in complete RPMI medium containing 100 mg/mL Liberase TH and 100 mg/mL DNase I at 37°C for 30 minutes under agitation. The cell suspension was then filtered through a 40 μm cell strainer and washed twice with cold PBS for further analysis.

### C. difficile colitis.

*C*. *difficile* infection of mice was performed as we previously described (33). Briefly, 8- to 10-week-old age- and sex-matched littermates were pretreated with antibiotic mixture (0.4 mg/mL kanamycin, 0.035 mg/mL gentamicin, 0.035 mg/mL colistin, 0.215 mg/mL metronidazole, and 0.045 mg/mL vancomycin; all from MilliporeSigma) added to the drinking water for 4 days. Next, mice received clindamycin (10 mg/kg, i.p.; MilliporeSigma). After 1 day, mice were infected with 10^8^ CFU *C*. *difficile* (VPI 10463 strain) by oral gavage. Mice were weighed and monitored daily during the entire protocol and assessed using a clinical severity score from 0 (normal) to 15 (33). Mice were sacrificed on day 5 of infection, and colons were collected for histology and IHC. Samples were analyzed in a blinded manner using histological scores as previously described (33).

### Translocation of C. difficile.

Spleens, livers, and mLNs were harvested on day 2 of *C*. *difficile* infection. Bacterial 16S rDNA was extracted using the PureLink Microbiome DNA Purification kit (Thermo Fisher Scientific) and quantified by qPCR using primers complementary to the eubacterium 16S rDNA conserved region. The bacterial load was determined by a standard curve with serial dilutions of *E*. *coli* genomic DNA, and CFU per gram of tissue were determined by dividing gene levels by sample weight as we previously described (33).

### Measurement of intestinal permeability with FITC-dextran.

On day 3 of *C*. *difficile* infection, mice received 200 μL FITC-dextran (MW 70,000; MilliporeSigma) suspension (250 mg/kg) by gavage. After 4 hours, mice were anesthetized, blood was collected by caudal puncture, and fluorescence readings were performed using a Multi-Mode Microplate Reader (Synergy HT) at 485/528 nm (excitation/emission). A standard curve was prepared with serial dilutions of FITC-dextran in PBS (33).

### C. rodentium infection.

Eight- to 10-week-old age- and sex-matched littermates were orally administered 2 × 10^9^ *C*. *rodentium*, strain DBS100 (American Type Culture Collection [ATCC]), as we previously described (28, 63). Body weights of mice were measured daily until day 10 or 20, when the mice were sacrificed for tissue collection. Samples were analyzed in a blinded manner using histology scores based on inflammation, edema, epithelial defects, crypt atrophy, and hyperplasia on a scale of 0 to 4 (64).

### DSS-induced colitis.

For acute colitis, mice received 3.5%–4% (w/v) DSS (MW 36,000–50,000, MP Biomedicals) in the drinking water for 7–8 days. Body weights and clinical scores were measured daily as previously described (17). For administration of IL-22, mice were injected i.p. with 1 μg recombinant mouse IL-22 daily.

### Adoptive T cell transfer–induced colitis.

Splenic CD4^+^CD45RB^hi^ T cells from WT mice were sort purified. T cells (5 × 10^5^) were transferred i.p. into cohoused *Ire1α^ΔRorc^* *Rag1^–/–^* and *Ire1α^fl/fl^* *Rag1^–/–^* mice. The mice were weighed every 5 days after transfer. The clinical score was graded on a scale of 0 to 5 and was based on decreased activity, hunched posture, ruffled fur, rate of breathing, shrunken eyes, shivering, rectal bleeding, and diarrhea as previously described (65, 66). The mice were euthanized on day 45 after transfer, and the colons were collected for measurement of length and histological analysis.

### Flow cytometry.

The following antibodies were used for flow cytometric analysis of mouse cells: CD3e (145-2C11, BioLegend), CD4 (RM4-5, BioLegend), CD5 (53–7.2, BioLegend), CD11b (M1/70, BioLegend), CD11c (N41B, Thermo Fisher Scientific), CD19 (6D5, BioLegend), CD45 (30-F11, BioLegend), CD90.2 (53-2.1, BioLegend), CCR6 (140706, BD Biosciences), B220 (RA3-6B2, BioLegend), Gr-1 (RB6-8C5, BioLegend), KLRG1 (2F1, BioLegend), NK1.1 (PK136, BioLegend), NKp46 (29A1.4, BioLegend), TER-119 (TER-119, BioLegend), RORγt (AFKJS-9, Thermo Fisher Scientific), FoxP3 (MF-14, BioLegend), GATA3 (L50-823, BD Biosciences), T-bet (eBio4B10, Thermo Fisher Scientific), Eomes (Dan11mag, Thermo Fisher Scientific), IL-22 (1H8PWSR, Thermo Fisher Scientific), IL-17A (TC11-18H10, BD Biosciences), and XBP1s (Q3-695, BD Biosciences). The following antibodies were used for flow cytometric analysis of human cells: CD3 (UCHT1, BioLegend), CD5 (UCHT2, BioLegend), CD11b (ICRF44, BioLegend), CD11c (Bu15, BioLegend), CD14 (QA18A22, BioLegend), CD19 (HIB19, BioLegend), CD45 (2D1, BioLegend), CD117 (104D2, BioLegend), CD127 (A019D5, BioLegend), NKp44 (P44-8, BioLegend), FcεRIα (AER-37, BioLegend), RORγt (REA278, Miltenyi Biotec), and IL-22 (2G12A41, BioLegend). Stained single-cell suspensions were analyzed on a FACSCanto II or a FACSymphony A3 cytometer (BD Biosciences). BD FACS ARIA II (BD Biosciences) was used for cell sorting. Analysis was performed using BD FACS Diva Software, version 8.0.1, and FlowJo, version 10 (FlowJo LLC).

### Generation and analysis of bulk RNA-Seq data.

Colonic LP cells were isolated from the colons of *Ire1α^ΔRorc^* and *Ire1α^fl/fl^* mice with acute DSS colitis. More than 5,000 ILC3s per mouse were sorted into RLT lysis buffer (provided in the QIAGEN RNeasy Plus Micro Kit). Total RNA was extracted using the QIAGEN RNeasy Plus Micro Kit. The RNA integrity number (RIN) was determined using the Agilent Bioanalyzer (Agilent Technologies). The RINs for all samples were greater than 9. Following the steps described by Peng and Cao et al. (62), in brief, cDNA was prepared using the SMARTer Ultra Low RNA kit for Illumina Sequencing (Takara-Clontech) at the Genome Technology Access Center (GTAC) at Washington University. cDNA was then fragmented and blunt ended. A base was added to the 3′ ends of cDNA fragments, and Illumina sequencing adapters were ligated to the ends. Ligated fragments were then amplified and sequenced using the Illumina HiSeq system. After base calling and demultiplexing, RNA-Seq reads were aligned to the mm10 genome. Aligned gene counts were processed using DESeq2 package (67) with R (version 4.3.2). Genes with fewer than 10 counts among all samples were excluded. For analysis of differentially expressed genes (DEGs), only protein-coding genes with an average expression of more than 100 counts per sample were used. The threshold of DEGs was set at an adjusted *P* value of less than 0.05 and a |log_2_ fold change| >1. Gene set enrichment analysis (GSEA) was performed using the clusterProfiler package (68) with R. Genes for GSEA were filtered as protein-coding genes with an average expression of more than 100 counts and an adjusted *P* value of less than 0.1.

### Analysis of scRNA-Seq data.

The count matrices and cell barcode–associated metadata were downloaded from Smillie et al. (https://www.ncbi.nlm.nih.gov/pmc/articles/PMC6662628/) (42). The cells annotated as “ILCs” were subsetted and processed separately using the Seurat R package. Specifically, only cells with unique molecular identifier (UMI) counts between 200 and 3,000 were kept, and cells whose percentage of mitochondrial genes were higher than 25% were discarded from the analysis. The resulting “ILCs” data set was then normalized and scaled using the “Sctransform” algorithm with regression of the percentage of mitochondrial genes. Standard principal component analysis (PCA) and uniform manifold approximation and projections (UMAPs) were run using 20 dimensions. Following this procedure, a DeSeq2-type analysis was performed to obtain the average expression of each gene in the “ILCs” cluster in each patient, for which purpose samples were first pseudobulked. Specifically, the resulting Seurat object was transformed into a SingleCellExperiment R object after running the function “DietSeurat” followed by “as.SingleCellExperiment,” and the matrix of log-transformed counts was generated by running the function “aggregate.Matrix” using the following parameters: groupings were defined by the author-determined metadata slots “sample” and “health,” which contained information about the sample ID and the health status of the patient who donated the sample, and the function was set to “sum.” Finally, an individual value for the log-transformed expression of each gene was obtained by computing the average between all biopsy sites taken from the LP of each patient identified with a unique patient ID. Represented are X healthy control samples, Y inflamed samples, and Z noninflamed samples.

### Statistics.

A 2-tailed Student’s *t* test or 1-way ANOVA with Tukey’s multiple-comparison test in GraphPad Prism 10.0 (GraphPad Software) was used for statistical analysis. A *P* value of less than 0.05 was considered statistically significant. Data represent the SEM or SD.

### Study approval.

All human studies were conducted under the approval of the IRBs of Washington University. Studies were conducted in accordance with the International Conference on Harmonization Good Clinical Practice Guideline, applicable regulations, and the Declaration of Helsinki. Patients provided written informed consent before enrollment. All animal studies were conducted under the approval of the IACUC of Washington University.

### Data availability.

The RNA-Seq data were deposited in the NCBI Gene Expression Omnibus (GEO) database (GEO GSE261091). All other data are reported in the Supporting Data Values file.

## Author contributions

SC conceptualized the study and designed experiments. SC, JLF, KM, QW, ZC performed mouse experiments and data analysis. SC, MAC, and PD were responsible for collection of human samples. SC and AUA analyzed human data. RJK provided reagents. SC and MC wrote the original draft of the manuscript. All authors reviewed and edited the manuscript. MC and SC acquired funding. MC supervised the study.

## Supplementary Material

Supplemental data

Supporting data values

## Version 1 05/09/2024

In-Press Preview

## Version 2 07/01/2024

Electronic publication
