# Supplementary material for: The IRE1α/XBP1 pathway sustains cytokine responses of group 3 innate lymphoid cells in inflammatory bowel disease
Source: J Clin Invest. 2024 May 9;134(13):e174198. doi: 10.1172/JCI174198 (PMC11214543; doi:10.1172/JCI174198)
Supplement: Supplemental data [file jci-134-174198-s008.pdf]

Supplemental Figure 1

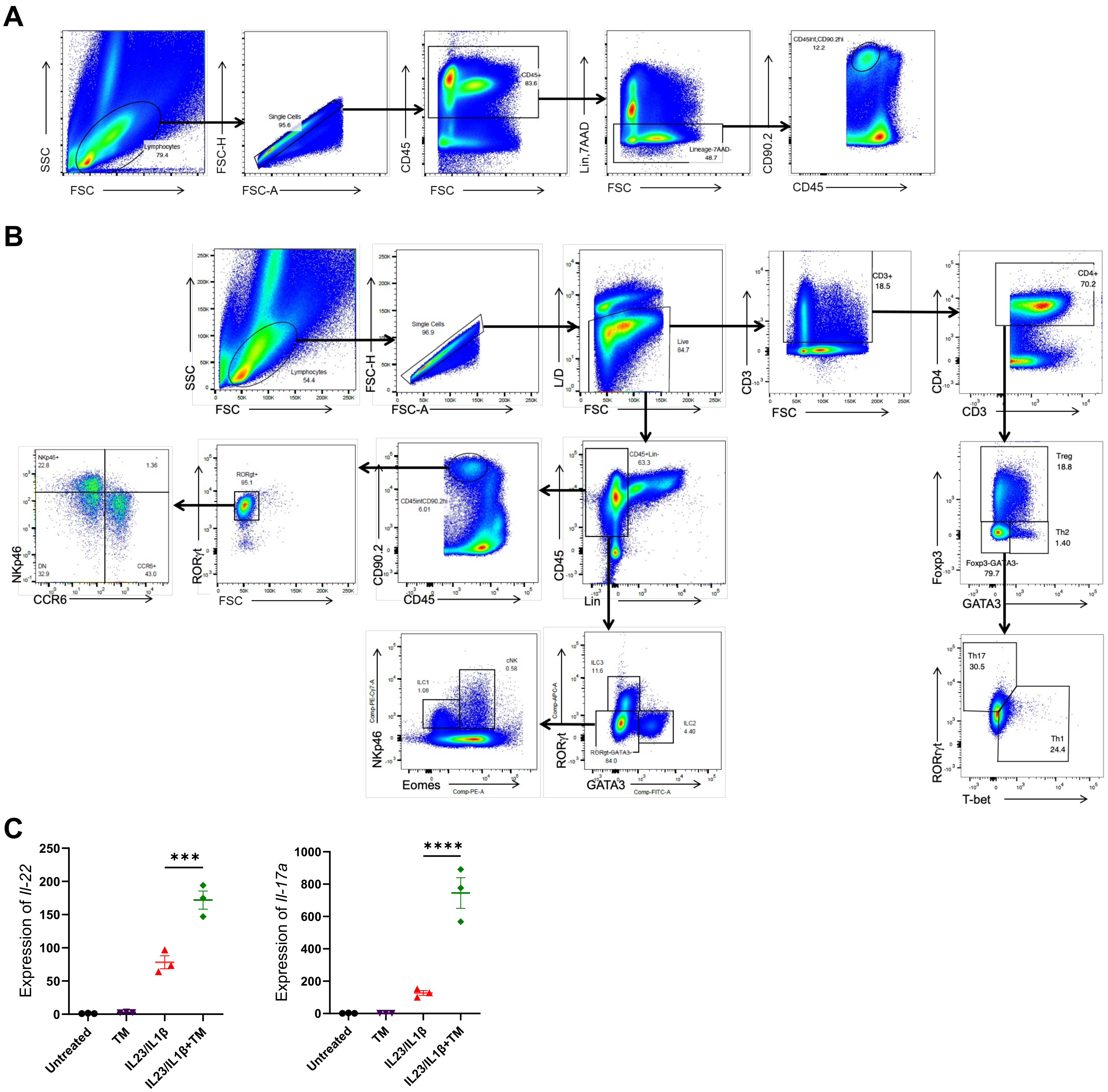

Supplemental Figure 1. Identification of mouse intestinal lymphocytes by flow cytometry and analysis of ILC3s by qPCR. (A) Sorting strategy of murine intestinal ILC3s, which were identified as live, lineage^{-}, CD45^{int}, CD90.2^{hi} lymphocytes. (B) Gating strategies for murine intestinal ILCs and T cells. (C) Sorted murine siLP ILC3s were untreated or treated with 1 ng/mL IL-23/IL-1β, 5 μg/mL tunicamycin (TM), or combination, as noted for 6h. Expression of cytokines was assessed by qPCR, relative to *β-actin* (n=3). P values were calculated using unpaired Student's t test (2-tailed) or one-way ANOVA with Tukey's multiple comparisons test. Error bars indicate SEM. *p<0.05, **p<0.01, ***p<0.001, ****p<0.0001.

Supplemental Figure 2

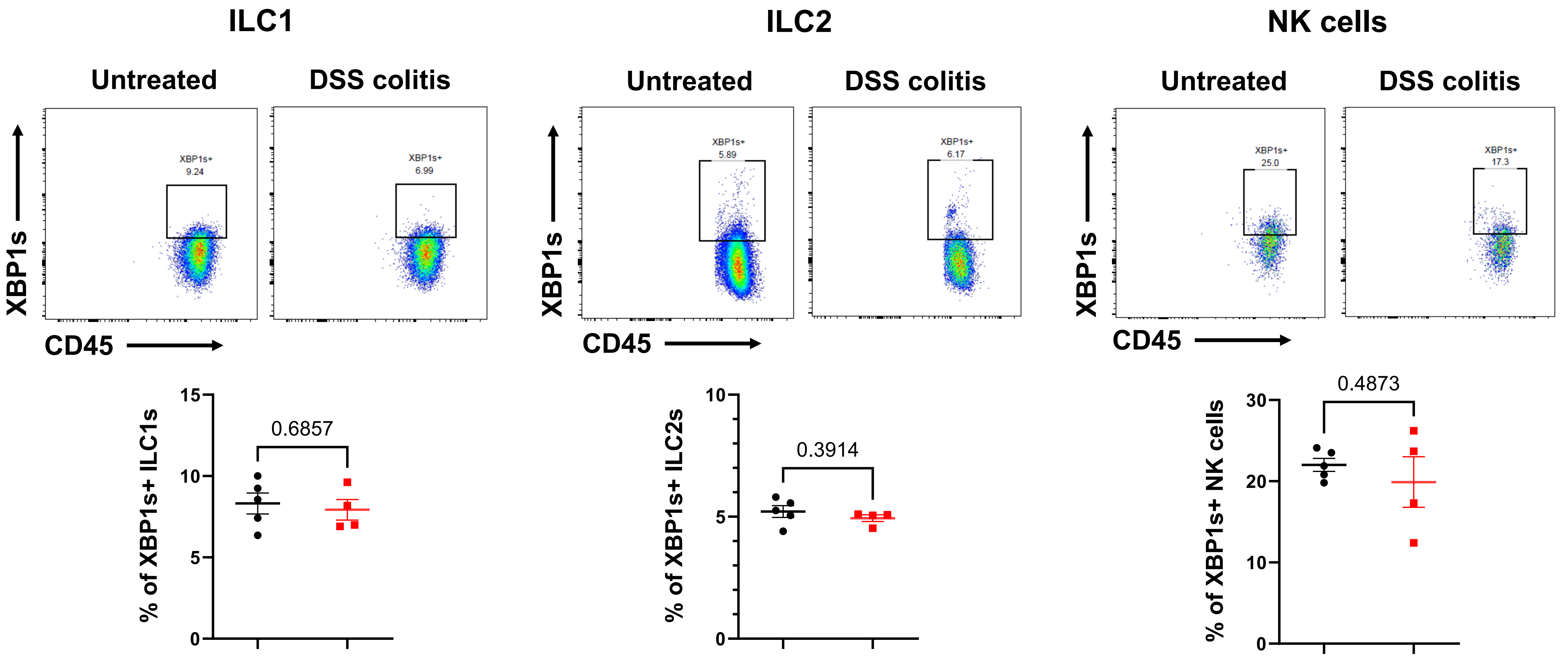

Supplemental Figure 2. The frequencies of XBP1s+ ILC1, ILC2, and NK cells are unchanged in mice with acute DSS colitis. Wild-type C57BL/6J mice were either untreated or given 3% DSS in drinking water for 5 days to induce acute colitis. Colonic ILC1, ILC2, and NK cells were identified by flow cytometry. The percentage of XBP1s+ cells from each mouse is shown. Error bars indicate SEM. P values were calculated using unpaired Student's t test (2-tailed).

Supplemental Figure 3

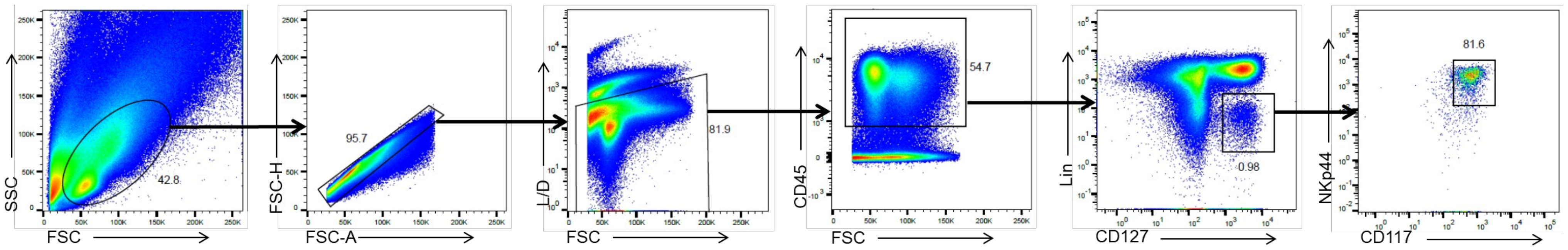

Supplemental Figure 3. Gating strategy for human intestinal ILC3s.

Supplemental Figure 4

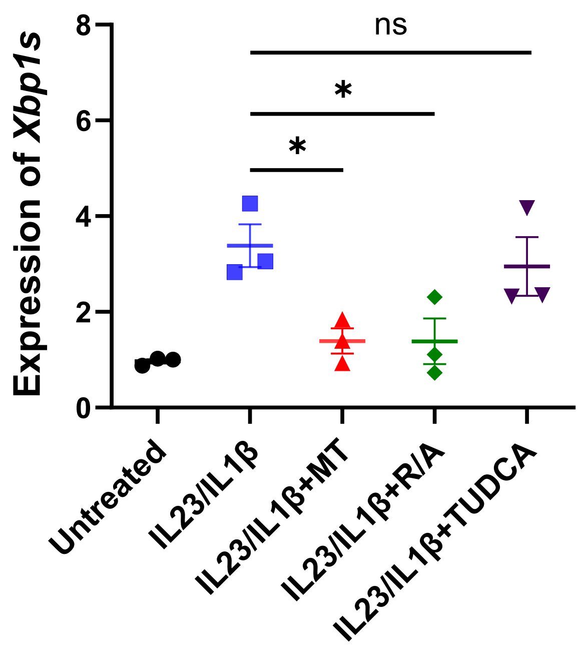

Supplemental Figure 4. Mitochondrial ROS is required for activation of *Xbp1s* by qPCR. Sorted siLP ILC3s were treated with IL-23/IL-1β, MitoTEMPO (MT), rotenone/antimycin A (R/A), or tauroursodeoxycholic acid (TUDCA) for 6h same as in Figure 2G. Expression of *Xbp1s* was assessed by qPCR. P values were calculated using one-way ANOVA with Tukey's multiple comparisons test. Error bars indicate SEM. *$p<0.05$.

Supplemental Figure 5

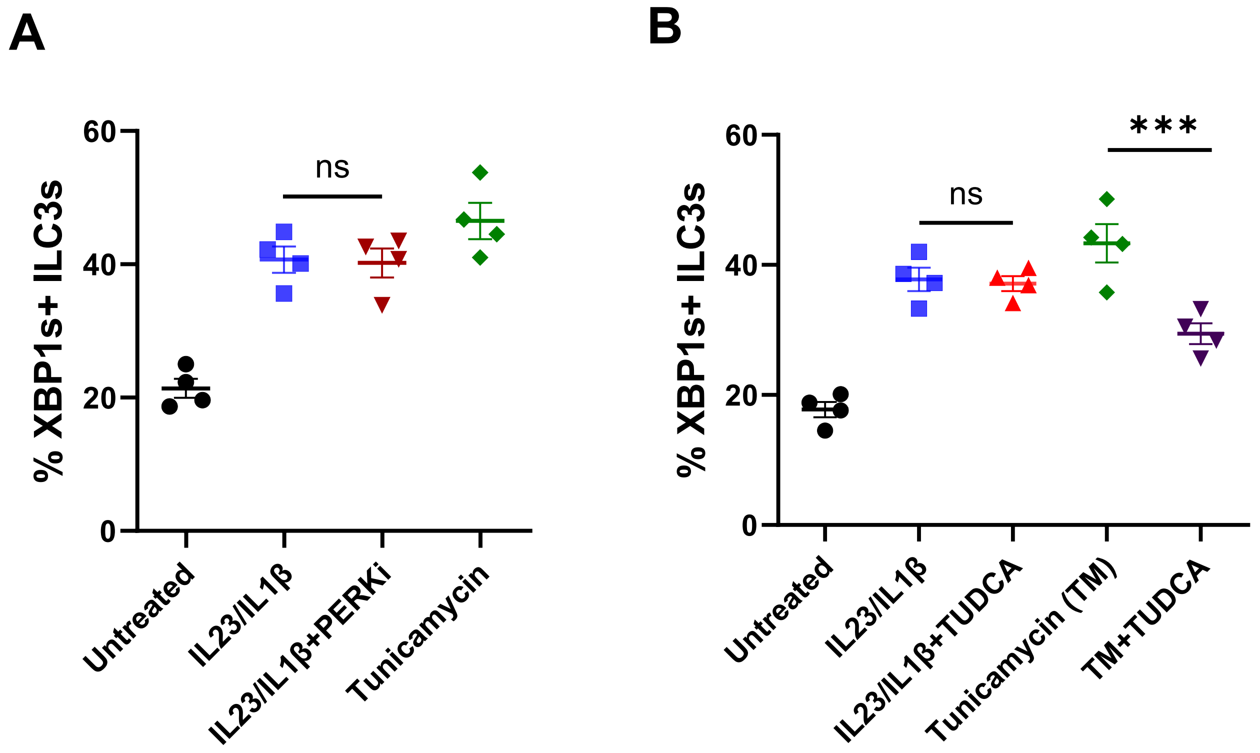

Supplemental Figure 5. Inhibition of the PERK pathway of UPR does not affect XBP1s in ILC3s; TUDCA reduces XBP1s induced by tunicamycin in ILC3s. Sorted siLP ILC3s were treated with 1 ng/mL IL-23/IL-1β, 2 μM PERK inhibitor (PERKi) GSK2606414, 5 μg/mL tunicamycin (TM), or 5 mM tauroursodeoxycholic acid (TUDCA) for 4h. Intracellular XBP1s was determined by flow cytometry. P values were calculated using one-way ANOVA with Tukey's multiple comparisons test. Error bars indicate SEM. ***p<0.001.

Supplemental Figure 6

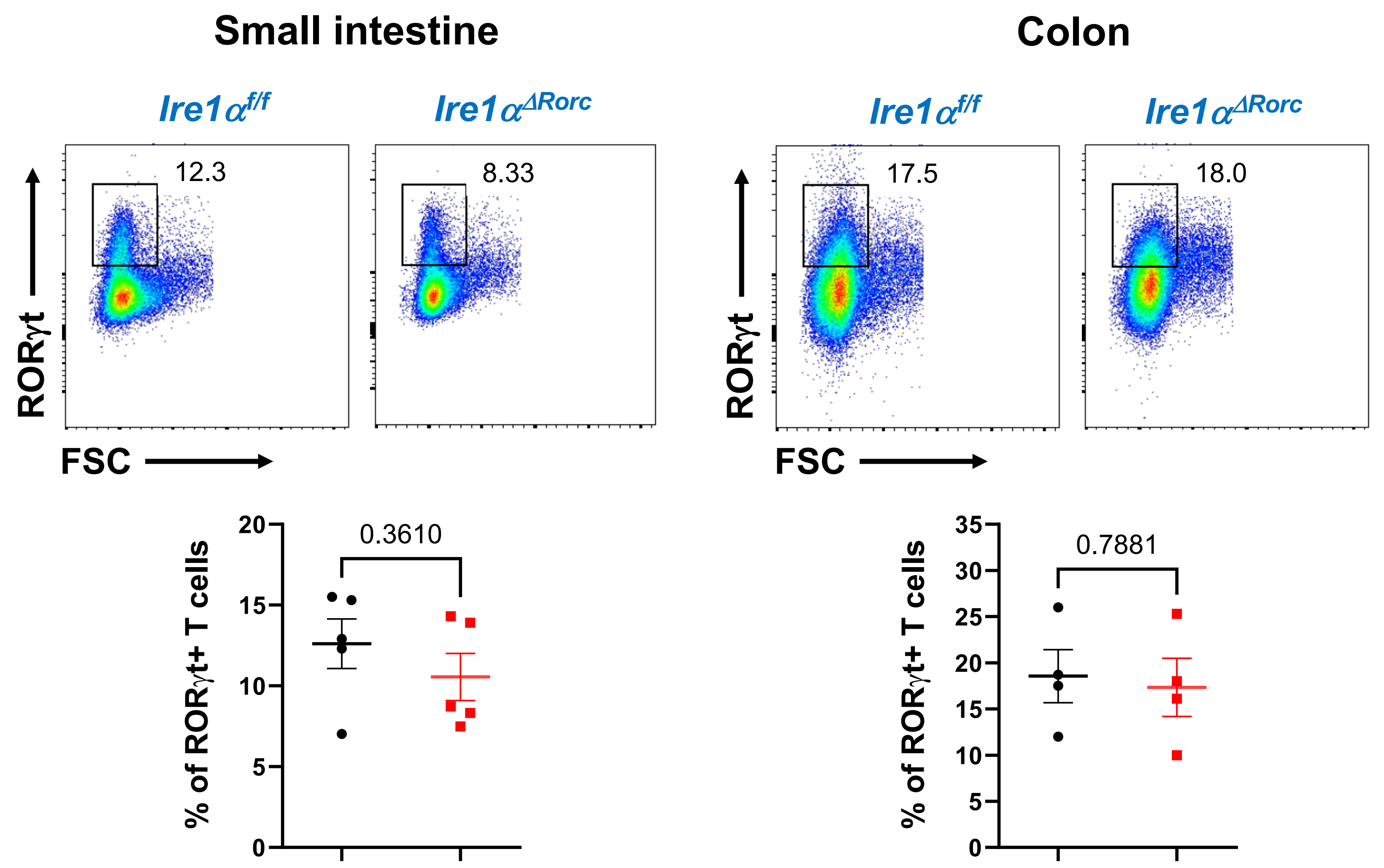

Supplemental Figure 6. The frequencies of RORγt+ T cells in the gut are unchanged in *Ire1α*^{ΔRorc} vs. *Ire1α*$^{flox/flox}$ (control) mice. Small intestinal and colonic LP cells were isolated from *Ire1α*^{ΔRorc} and *Ire1α*$^{flox/flox}$ (control) mice, RORγt+ T cells were identified using flow cytometry. P values were calculated using unpaired Student's t test (2-tailed).

Supplemental Figure 7

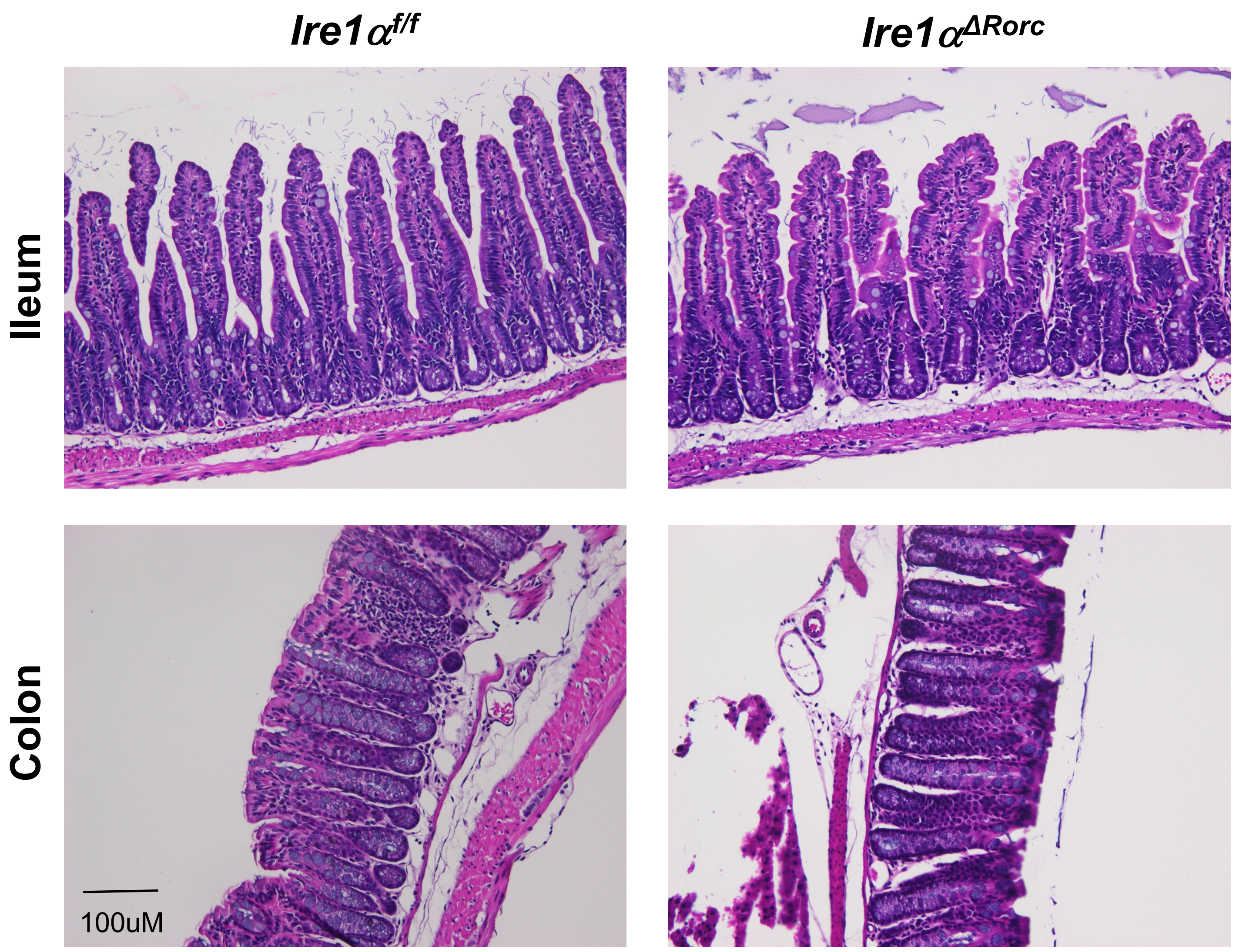

Supplemental Figure 7. The ileum and colon of *Ire1α^{ΔRorc}* mice are morphologically unchanged at steady state. The ilea and colons of *Ire1α^{ΔRorc}* and *Ire1α$^{flox/flox}$* (control) mice were analyzed by H&E staining.

Supplemental Figure 8

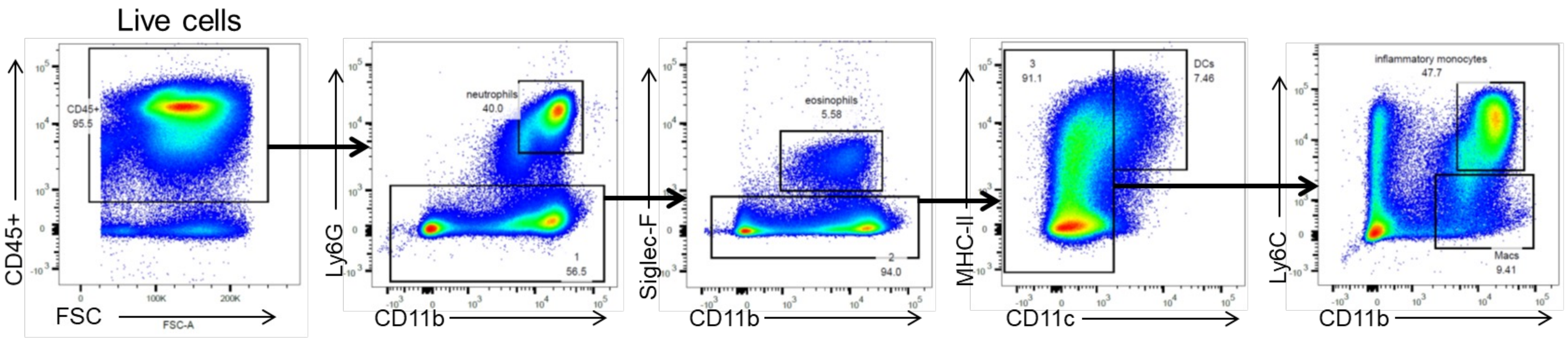

Supplemental Figure 8. Gating strategy for mouse myeloid cells in the colon.

Supplemental Figure 9

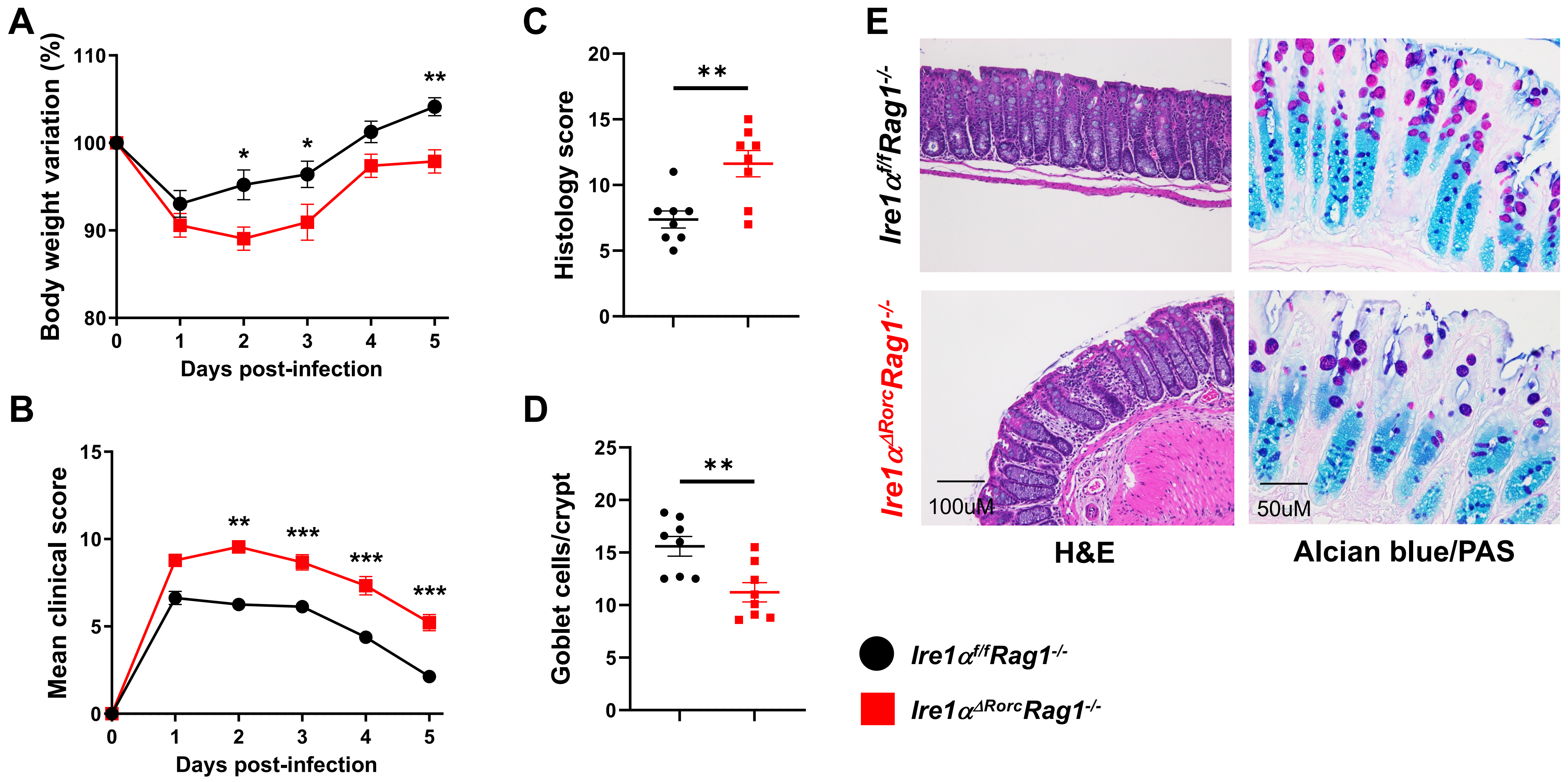

Supplemental Figure 9. *Ire1α$^{\Delta Rorc}$Rag1$^{-/-}$* mice are more susceptible to acute *C. difficile* infection. *Ire1α$^{\Delta Rorc}$Rag1$^{-/-}$* and *Ire1α$^{f/f}$Rag1$^{-/-}$* (control) mice were orally infected by *C. difficile* following antibiotic treatment. Weight loss (A) and clinical scores (B) were measured daily throughout the course of infection (n = 8 or 9). (C-E) Mice were sacrificed on the day 5 post-infection and histology were assessed. P values were calculated using unpaired Student t test (2-tailed). Numbers indicate means ± SEM. *p<0.05, **p<0.01, ***p<0.001.

Supplemental Figure 10

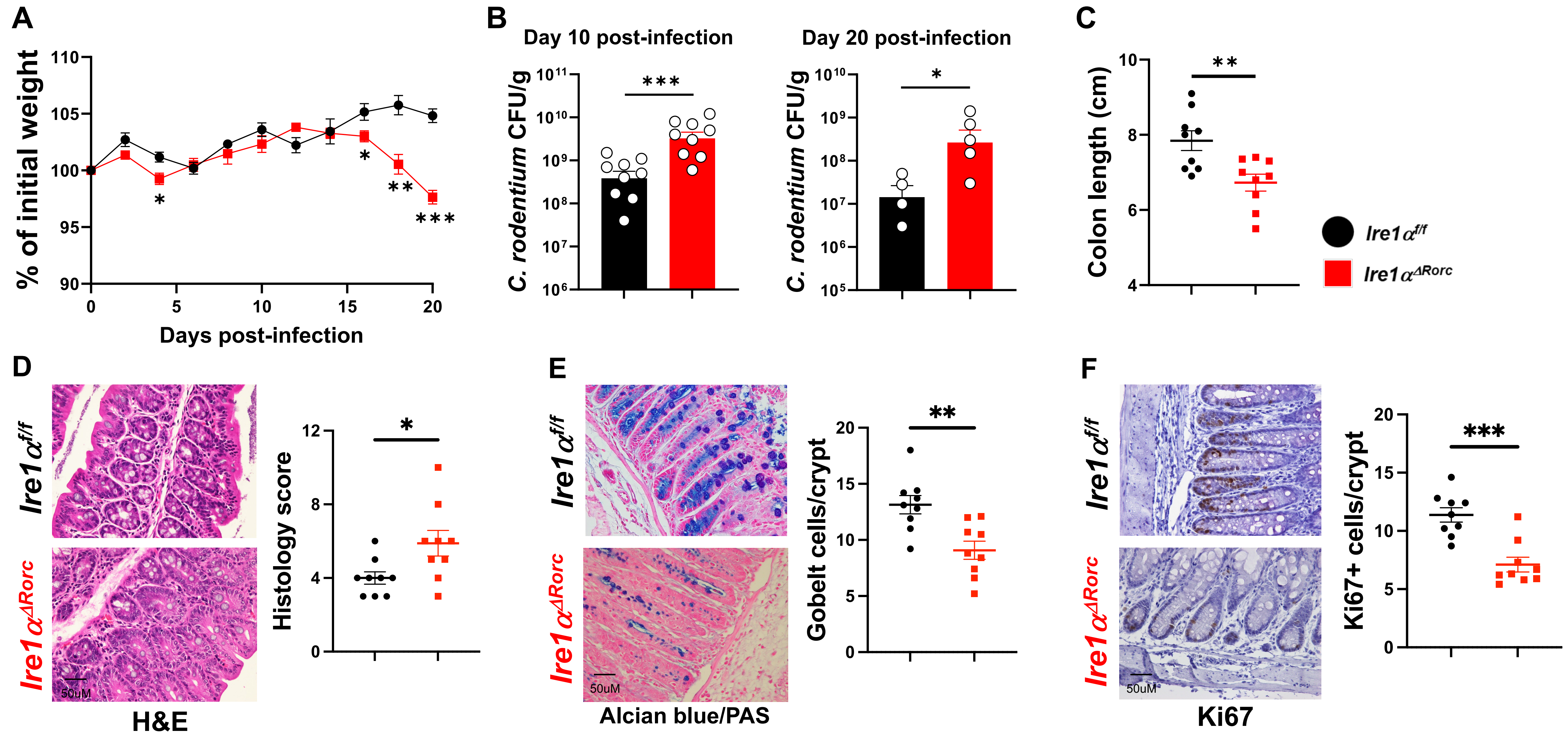

Supplemental Figure 10. *Ire1α^{ΔRorc}* mice are more susceptible to *C. rodentium* infection. *Ire1α^{ΔRorc}* and *Ire1α$^{flox/flox}$* (control) mice were orally infected by *C. rodentium*. (A) weight loss was measured daily throughout the course of infection (n = 9). (B-F) Mice were sacrificed on the day 10 or 20 post-infection, CFU of *C. rodentium*, colon length (day 10), and histology (day 10) were assessed. P values were calculated using unpaired Student t test (2-tailed). Numbers indicate means ± SEM. *p<0.05, **p<0.01, ***p<0.001.

Supplemental Figure 11

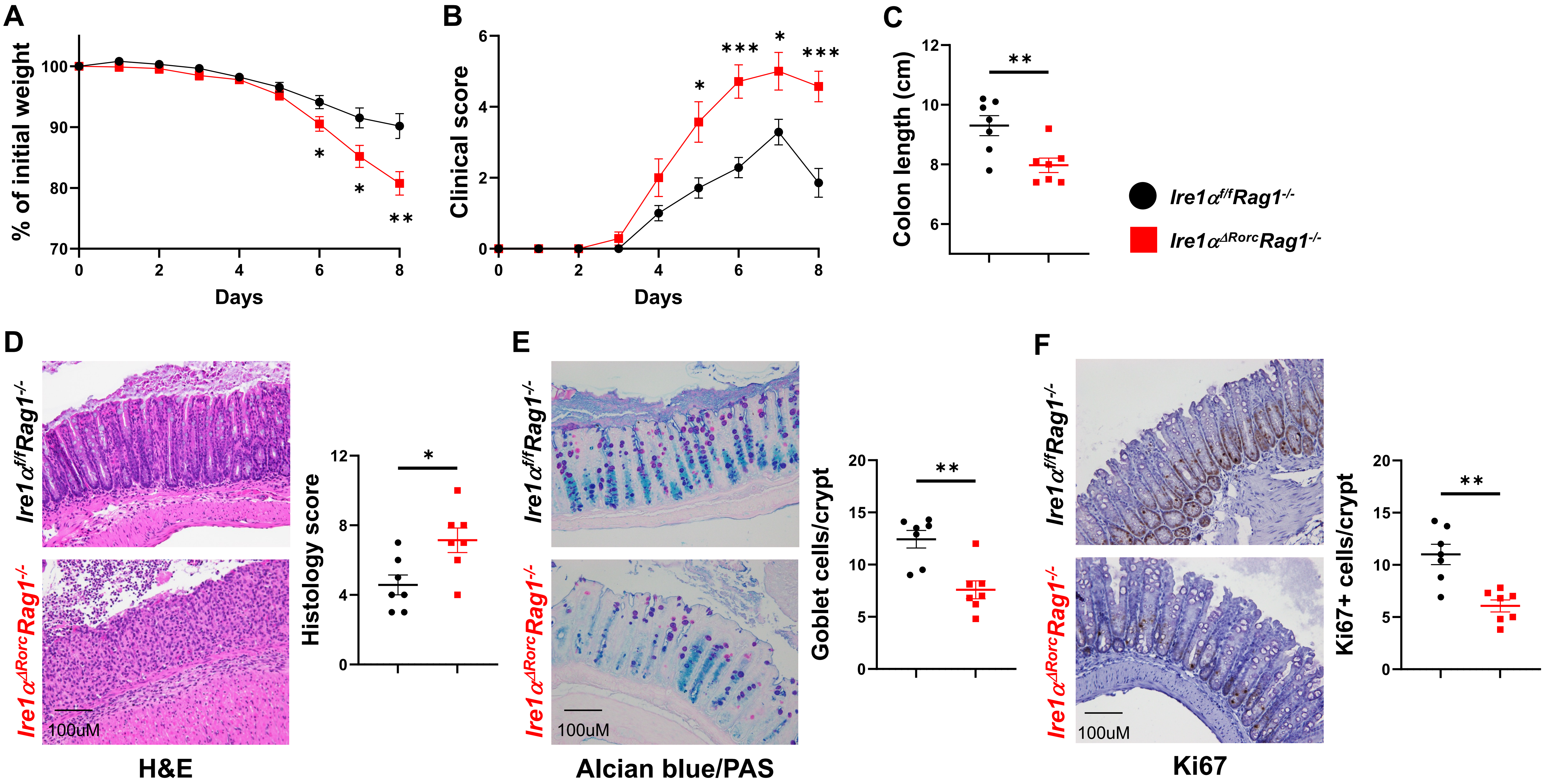

Supplemental Figure 11. *Ire1α$^{\Delta Rorc}$Rag1$^{-/-}$* mice are more susceptible to acute DSS colitis. *Ire1α$^{\Delta Rorc}$Rag1$^{-/-}$* and *Ire1α$^{f/f}$Rag1$^{-/-}$* (control) littermates given 3.5% DSS in drinking water for 7 days followed by one day of fresh water. Weight loss (A) and clinical scores (B) were measured daily throughout the course (n = 7). (C-F) Mice were sacrificed on the day 8, colon length and histology were assessed. P values were calculated using unpaired Student t test (2-tailed). Numbers indicate means ± SEM. *p<0.05, **p<0.01, ***p<0.001.

Supplemental Figure 12

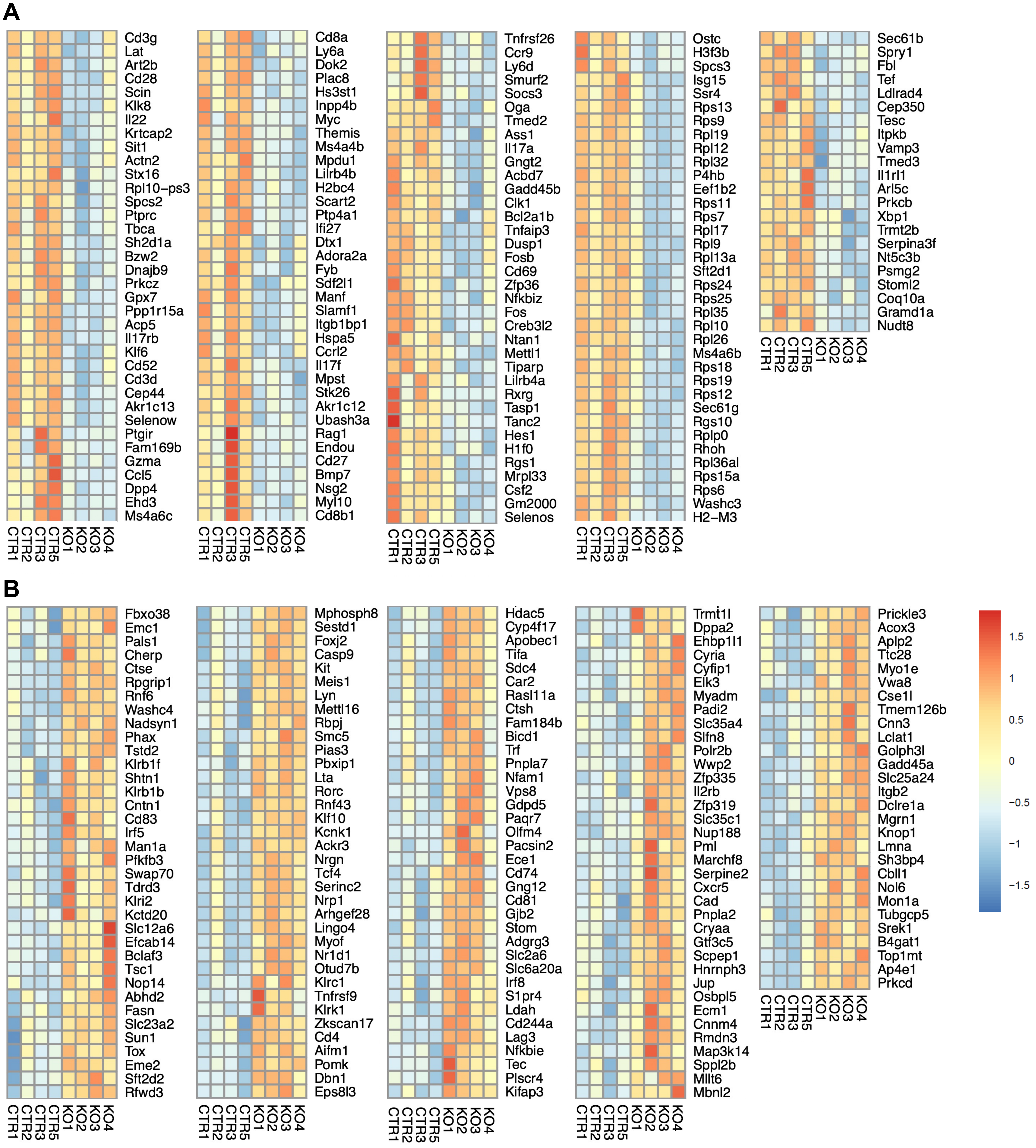

Supplemental Figure 12. Differentially expressed genes by bulk RNA-seq. Downregulated (A) and upregulated (B) genes in colonic ILC3s from *Ire1α*$^{\Delta Rorc}$ vs. *Ire1α*$^{f/f}$ mice with acute DSS colitis.

Supplemental Figure 13

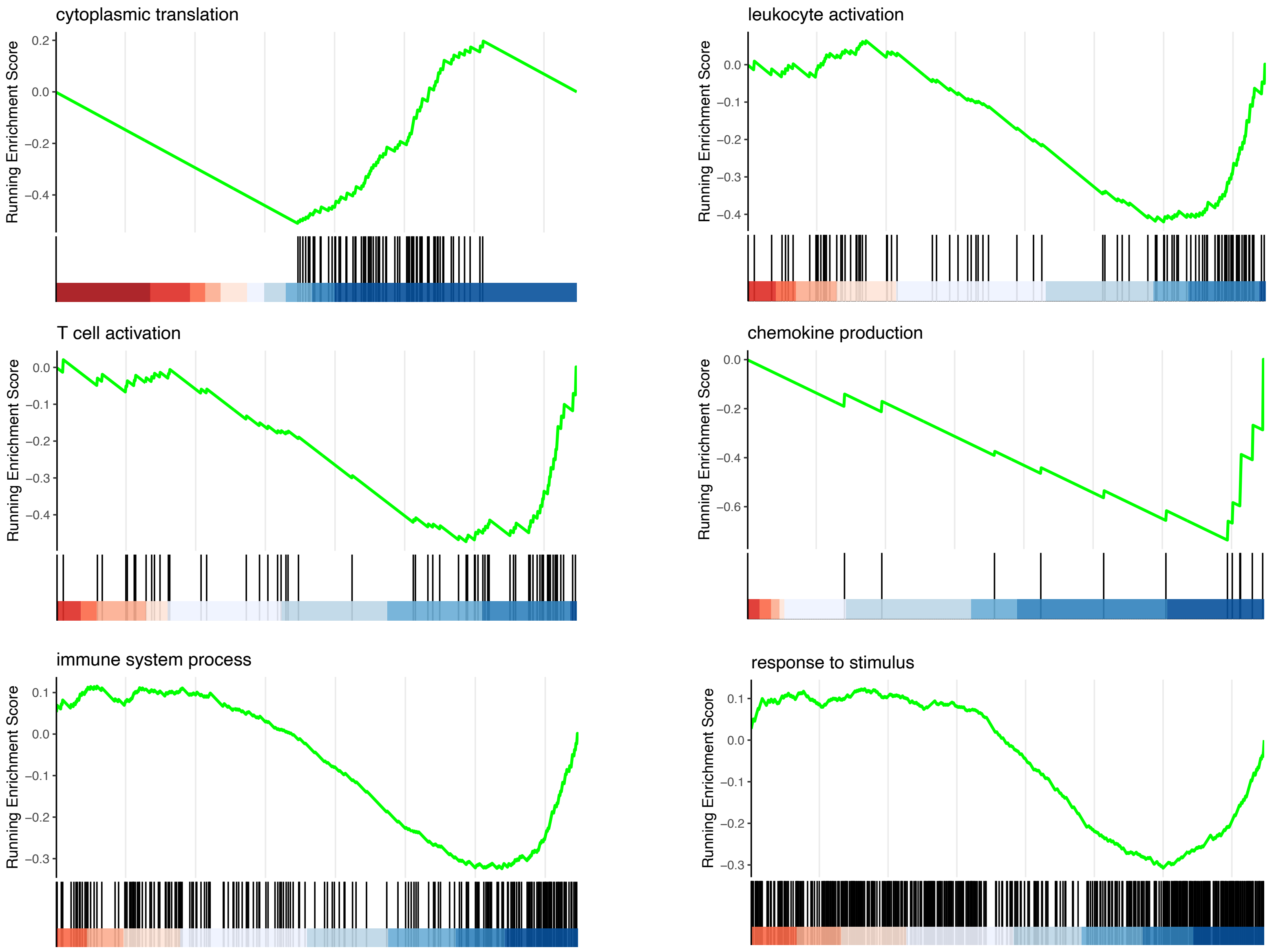

Supplemental Figure 13. Pathway analysis of gene expression. Analysis of selected pathways in colonic ILC3s from *Ire1*$\alpha^{\Delta Rorc}$ vs. *Ire1*$\alpha^{f/f}$ mice with acute DSS colitis.

Supplemental Figure 14

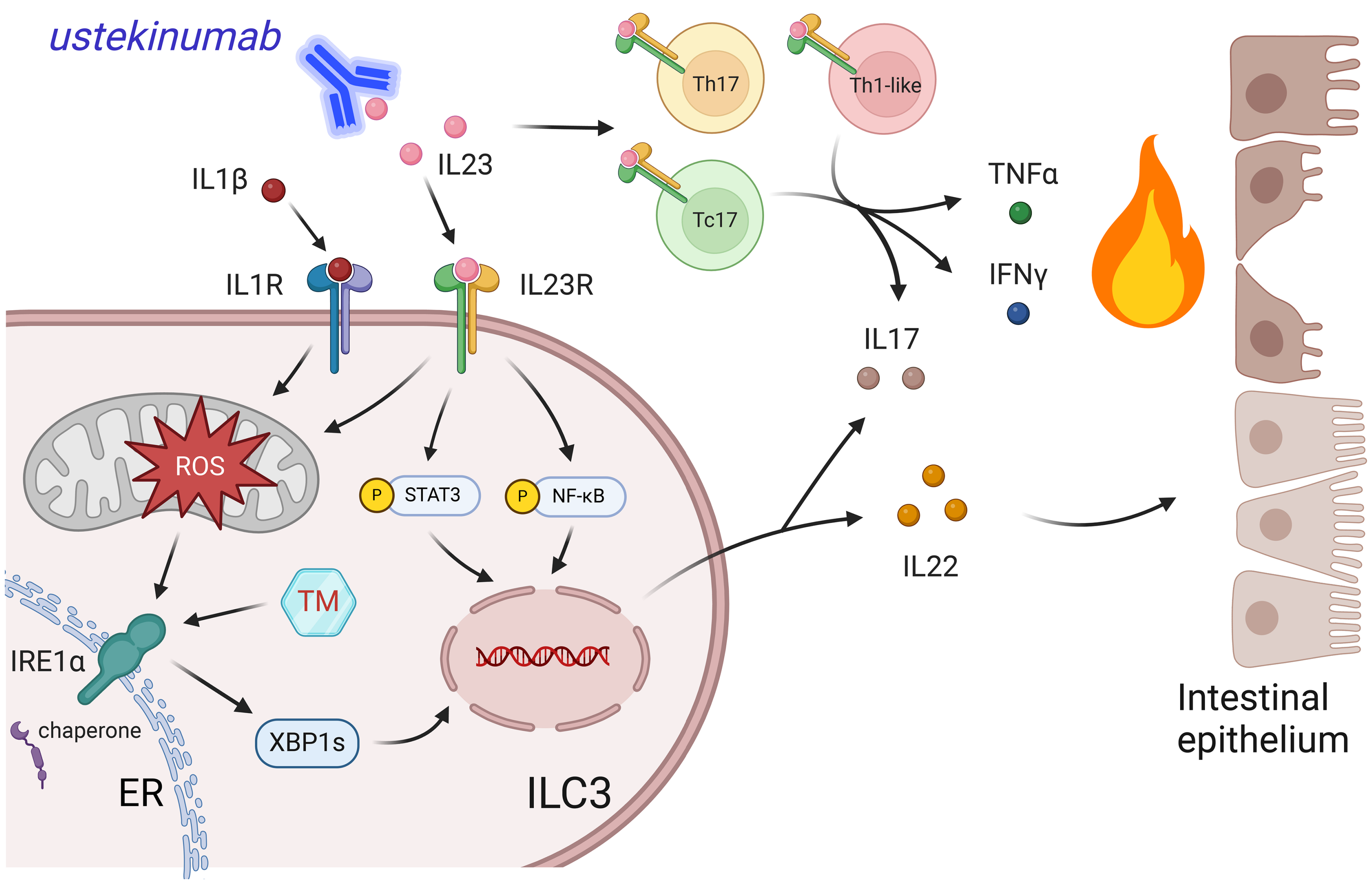

Supplemental Figure 14. Graphical abstract (prepared using BioRender).

Supplemental Methods

Quantitative real-time PCR

cDNA was synthesized from mouse RNA with Superscript III first-strand synthesis system for qPCR (Invitrogen). RNA expression was analyzed by qPCR using Universal SYBR Green PCR Master Mix (Bio-Rad Laboratories) and an ABI7000 (Applied Biosystems). The following primers (5’ to 3’) were used:

Xbp1s (f): GAGTCCGCAGCAGGTG
Xbp1s (r): GTGTCAGAGTCCATGGGA
Pdi (f): CAAGATCAAGCCCCACCTGAT
Pdi (r): AGTTCGCCCCAACCAGTACTT
Bip (f): TCATCGGACGCACTTGGA
Bip (r): CAACCACCTTGAATGGCAAGA
Grp94 (f): AATAGAAAGAATGCTTCGCC
Grp94 (r): TCTTCAGGCTCTTCTTCTGG
Chop (f): GTCCCTAGCTTGGCTGACAGA
Chop (r): TGGAGAGCGAGGGCTTTG
Il17a (f): AGCTCCAGAAGGCCCTCAGACTACC
Il17a (r): GACTTTGAGGTTGACCTTCACAT
Il22 (f): AGAATGTCAGAAGGCTGAAGG
Il22 (r): CAGCTTTCCCTCCGCATTGACAC
Muc1 (f): CCCTACCTACCACACTCACGGACG
Muc1 (r): GTGGTCACCACAGCTGGGTTGGT
Muc2 (f): CGACTGTGAGCAGTGTGTCA
Muc2 (r): GGGTAGGGTCACCTCCATCT
Reg3g (f): AACAGAGGTGGATGGGAGTG
Reg3g (r): GGCCTTGAATTTGCAGACAT
Gapdh (f): TTCAACGGCACAGTCAAGG
Gapdh (r): CATGGACTGTGGTCATGAG
β-actin (f): TGTTACCAACTGGGACGACA
β-actin (r): CTGGGTCATCTTTTCACGGT
18s (f): CGCTTCCTTACCTGGTTGAT
18s (r): GAGCGACCAAAGGAACCATA
16s of eubacteria (f): ACTCCTACGGGAGGCAGCAGT
16s of eubacteria (r): ATTACCGCGGCTGCTGGC
